# Linking migration and microbiota at a major stopover site in a long-distance avian migrant

**DOI:** 10.1186/s40462-022-00347-0

**Published:** 2022-11-07

**Authors:** Nikki Thie, Ammon Corl, Sondra Turjeman, Ron Efrat, Pauline L. Kamath, Wayne M. Getz, Rauri C. K. Bowie, Ran Nathan

**Affiliations:** 1 Movement Ecology Lab, The Hebrew University of Jerusalem, Jerusalem, Israel; 2 Museum of Vertebrate Zoology, University of California, Berkeley, Berkeley, CA USA; 3 School of Food and Agriculture, University of Maine, Orono, ME USA; 4 Department of Environmental Science Policy and Management, University of California, Berkeley, Berkeley, CA USA; 5 School of Mathematical Sciences, University of KwaZulu-Natal, Durban, South Africa; 6 Department of Integrative Biology, University of California, Berkeley, Berkeley, CA USA; 7 Present Address: Azrieli Faculty of Medicine, Bar-Ilan University, Safed, Israel; 8 Present Address: Mitrani Department of Desert Ecology, Jacob Blaustein Institutes for Desert Research, Ben-Gurion University of the Negev, Midreshet Ben-Gurion, Israel

**Keywords:** Cloacal microbiota, Long-distance migration, Steppe buzzard, Stopover bottleneck, GPS-tracking

## Abstract

Migration is one of the most physical and energetically demanding periods in an individual bird’s life. The composition of the bird’s gut or cloacal microbiota can temporarily change during migration, likely due to differences in diets, habitats and other environmental conditions experienced *en route*. However, how physiological condition, migratory patterns, and other drivers interact to affect microbiota composition of migratory birds is still unclear. We sampled the cloacal bacterial microbiota of a long-distance migrant, the steppe buzzard (*Buteo buteo vulpinus*), at an important spring stopover bottleneck in Eilat, Israel, after crossing the ca. 1800 km Sahara Desert. We examined whether diversity and composition of the cloacal microbiota varied with body condition, sex, movement patterns (i.e., arrival time and migration distance), and survival. Early arrival to Eilat was associated with better body condition, longer post-Eilat spring migration distance, higher microbial α-diversity, and differences in microbiota composition. Specifically, early arrivals had higher abundance of the phylum Synergistota and five genera, including *Jonquetella* and *Peptococcus*, whereas the phylum Proteobacteria and genus *Escherichia-Shigella* (as well as three other genera) were more abundant in later arrivals. While the differences in α-diversity and *Escherichia-Shigella* seem to be mainly driven by body condition, other compositional differences associated with arrival date could be indicators of longer migratory journeys (e.g., pre-fueling at wintering grounds or stopover habitats along the way) or migratory performance. No significant differences were found between the microbiota of surviving and non-surviving individuals. Overall, our results indicate that variation in steppe buzzard microbiota is linked to variation in migratory patterns (i.e., capture/arrival date) and body condition, highlighting the importance of sampling the microbiota of GPS-tracked individuals on multiple occasions along their migration routes to gain a more detailed understanding of the links between migration, microbiota, and health in birds.

**Supplementary Information:**

The online version contains supplementary material available at 10.1186/s40462-022-00347-0.

## Background

Many bird species spend a significant part of their annual cycle migrating [1, 2], flying thousands of kilometers between favorable breeding- and non-breeding locations while coping with variable flight conditions and foraging habitats *en route* [3]. Long-distance migration is physiologically and energetically demanding and involves morphological and physiological adjustments, such as shifts in flight muscles, body fat, and overall body mass [4], as well as downregulation of digestion [5] or immune response [6]. The physiological adjustments associated with migration could be linked to the microbiota composition found in an individual’s gut, known to play an important role in processes like digestion and nutrient uptake [7], immune system function [8] and protection against pathogenic infections [9]. Host-associated microbiota are influenced by various intrinsic (e.g. genetics, physiology, sex [10, 11]) and extrinsic factors (e.g. habitat, behavior, diet [12–14]). The physical challenges and changing environments experienced during migration could thus be accompanied by important changes and/or beneficial adjustments in migratory birds’ microbiota.

Recent studies have suggested that bird migration can indeed affect the microbial diversity and composition found in birds (e.g., [15–18]). However, these findings mainly highlight (but did not directly test) the role of diet-related environmental conditions. For example, resampling the same Kirtland’s warbler (*Setophaga kirtlandii*) individuals revealed that their fecal microbiota significantly differs between wintering and breeding grounds, yet remains similar over two months at the breeding grounds [18]. Fecal and cloacal microbiota composition, respectively, have also been found to differ between migrant and resident barn swallows (*Hirunda rustica*) [17], curlew sandpipers (*Calidris ferruginea*) and red-necked stints (*Calidris ruficollis*) [16, 19]. Additionally, bacterial communities were more diverse among individual migrant barn swallows compared to their resident counterparts, potentially due to migrants being exposed to a greater diversity of bacteria either at their different breeding areas or various stopover sites visited during migration [17]. High inter-individual variation in gut microbiota (measured from feces) has also been observed in other migratory passerine species and variation has been found to decrease with time spent at a shared stopover site (observed in Swainson’s thrush (*Catharus ustulatus*), wood thrush (*Hylocichla mustelina*) and gray catbird (*Dumetella carolinensis*) [15, 20]), or at the breeding grounds (observed in Kirtland’s warbler [18]). Altogether, these studies highlight the substantial variation in microbiota composition in migratory birds, which is typically hypothesized to be caused by exposure to new bacteria when migrants change environments or their diets [15, 17, 18, 20].

Previous studies have shown that physiological stress can decrease microbial diversity [21] and both stress and poor body conditions have been connected to microbial dysbiosis [15, 22]. Changes in microbial diversity and community dysbiosis have also been associated with lower survival rates [23–26]. Further, although age- and sex-related variation in microbiota composition is commonly reported for birds (e.g., [14, 24, 27, 28]), details about such effects are still missing in the context of bird migration. Likewise, only a few studies investigating the microbiota of migratory birds have incorporated important drivers like physiological condition [15], whereas no studies to date have included detailed information about the migration routes or timing of microbiota-sampled birds, or have compared the microbiota of individuals that completed their migration journey versus those that died along the way.

To address these knowledge gaps, we investigated how cloacal microbiota composition is linked to different aspects of migratory movements and performance (i.e., body condition, arrival time to stopover site, movement patterns, and survival) in migrating steppe buzzards (*Buteo buteo vulpinus*). Migratory connectivity in this long-distance migrant has been inferred from ring recoveries of birds that (likely) bred across a wide range (20–100° E, 35–65° N) of the Palearctic region and wintered in southern Africa [29–31], suggesting large inter-individual variation in migration routes (total distances range from 9200 to 14,200 km [32]). Along with millions of other migratory birds, steppe buzzards migrate through the important migratory bottleneck in the southern Arava of Israel during spring migration [33, 34], right after crossing a large ecological barrier, the Sahara Desert. More detailed information on their migration routes (e.g. from GPS tracking), however, has been lacking thus far. Previous studies revealed that adult buzzards arrive in the southern Arava earlier than juveniles and that early arriving individuals of all ages and sexes are in better physiological state than late arrivals [33]. The possibility for capture, sampling and tracking of individuals that follow different migration routes that funnel through a single major stopover site makes the steppe buzzard a promising model species to investigate microbiota-migration links.

We examined several hypotheses on the links between bacterial microbiota and migration by combining GPS tracking and microbiota sampling. Specifically, we considered how variation in age, sex, physiological condition, arrival time and survival are linked to cloacal microbiota composition in steppe buzzards. Disentangling the independent role of each of these potential drivers is constrained by known innate correlations among age, body condition and arrival time, further supported by our data. We thus focused on the following three main hypotheses. First, since sex-related differences in microbiota composition have previously been observed in bird species showing other sex-related physiological or behavioral differences [14, 27, 28], we expect the microbial community to differ between male and female buzzards (H-1) due to the notable sexual size dimorphism in raptor species [35]. Second, given that body condition of birds has been associated with changes in microbiota diversity and composition [15, 36], and that average body condition tends to differ with arrival date at stopovers [33, 37, 38] (H-2a), we expect that early arriving individuals will differ from later-arriving individuals in microbiota diversity and composition (H-2b). Third, because body condition is a strong predictor of migration survival [39–42], we expect individuals with poorer body conditions to have a different microbiota diversity and composition (H-3a), and similarly, individuals that died during migration will differ in their microbiota diversity and composition from those that survived (H-3b).

## Methods

### Study system and sample collection

The steppe buzzard (*Buteo buteo vulpinus*) is a subspecies of the abundant common buzzard (*Buteo buteo buteo*) [43]. They are opportunistic predators that are mainly associated with open country or edges of woodland areas, where they mainly feed on small mammals but also birds, reptiles, and invertebrates [35, 43, 44]. Steppe buzzards breed throughout the northern Palearctic region, ranging from eastern Europe to central or western Asia, and winter throughout southern Africa [29–31, 43], and during migration seasons pass in large numbers through the Middle East, especially in the Southern Arava [33, 34].

**Fig. 1:**
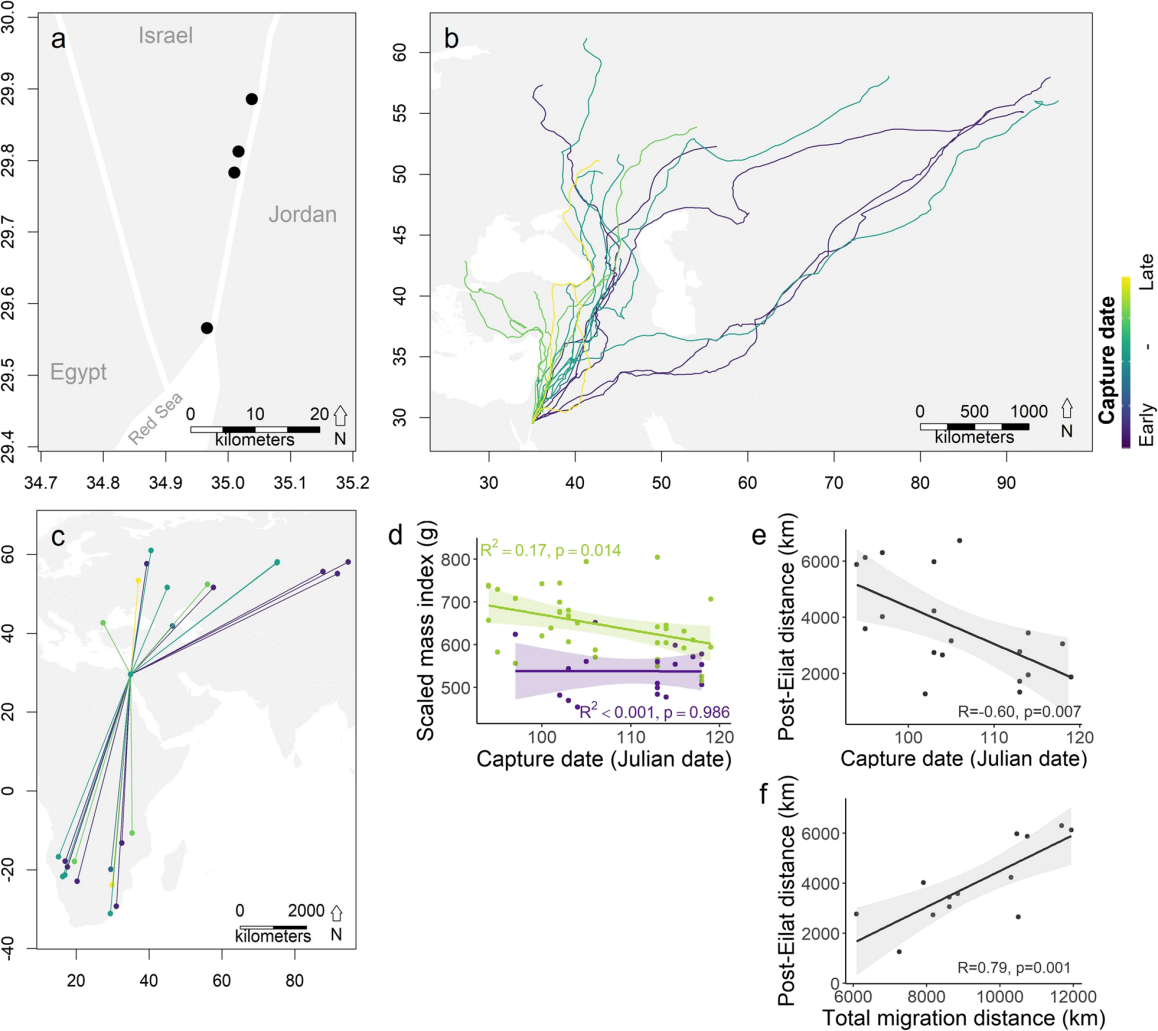
Association of steppe buzzard capture date with body condition and migration distances. **a** Capture locations in the southern Arava, Israel. **b** Post-Eilat spring migration routes to breeding grounds and **c** total migration connectivity for GPS-tagged individuals that survived an entire spring migration or until the next wintering season, respectively. The routes are colored by capture date, ranging between earlier arrival in purple to later arrival in yellow. **d** Capture (Julian) date was negatively correlated with body condition based on a scaled mass index (SMI) for females (green), but not for males (purple). **e** Capture date was negatively related to post-Eilat migration distance. **f** Total migration distance was positively related to post-Eilat migration distance

We trapped steppe buzzards in April 2019 during spring migration in the southern Arava region of Israel (Fig. 1a). A total of 54 steppe buzzards (36 females, 18 males) were caught using bal-chatri traps [45], from which they were immediately extracted and kept in individual cloth bags for up to 30 min until processing. Captured individuals were ringed, measured (tarsus and wing length), weighed and aged (EURING age codes [46]) based on plumage [47], followed by sample collection for bacterial community characterization (cloacal swab, stored in sterile 1.5 mL centrifuge tubes with 1 mL 95% EtOH) and genetic sex determination (3 chest feathers, stored dry [17, 48]). Samples were kept frozen at − 20 °C in temporary storage in the field for up to 5 days until reaching the laboratory for long-term storage at − 80 °C.

### GPS tracking

In addition to sample collection, a total of 37 buzzards (30 females, 7 males) were fitted with 20- or 25 g solar-powered GPS tags (Ornitela, Lithuania) attached in leg-loop formation. The GPS tags were only fitted on individuals weighing > 550 g, such that the tag plus harness (weighing 24 or 29 g) did not exceed the generally recommended 5% (mean ± s.d.: 4.0 ± 0.4%) of the individual’s body mass to reduce the possibility of introducing adverse effects on the individuals’ behavior and survival caused by the additional weight [49]. The tags recorded GPS positions and acceleration (ACC) data once every 10–20 min. Sampling frequency was reduced to once every 3 h when battery levels dropped below 25%.

### Demographics and body condition

Individuals were split into two categories, juvenile (1st year: EURING age 5) or adult (2nd year and older: EURING age ≥ 6). Despite the strong sexual dimorphism in steppe buzzards, there is still a considerable overlap in body mass and size [33]; we measured a body mass of 629 ± 77 g for females (*n* = 36) and 518 ± 60 g for males (*n* = 18). Therefore, a genetic assay was used to assign sex (as in [17] but using the 2550 forward and 2718 reverse primers described in [50]). Body mass and wing length were used to calculate a scaled mass index (SMI: standardizing mass to the arithmetic mean of wing length) related to body condition [51]. SMI was calculated separately for female and male individuals due to the strong sexual dimorphism in steppe buzzards, which may affect the relationship between morphometrics and true body condition (i.e., fat load vs. lean mass) for each sex. For the same reason, subsequent analyses including body condition as a variable were performed for females and males separately.

### Movement analysis

The dataset was filtered to only include spring migration tracks of each individual. For this, we subsampled to 60 min intervals between sunrise and sunset of each day and calculated daily movement characteristics including total distance traveled, beeline distance (measured by rhumb line) between roosting sites (i.e., start and end of each day), and directionality of beeline and turning angle compared to the previous day. Arrival to the breeding/summer area was characterized by a cessation in long-distance daily movements (> 50 km) in a general northern direction (azimuth > 270° and < 90°) and an increased turning angle compared to the track of the previous day (> 100°), which was validated by visual inspection of the tracks. Alternative migration endpoints were death (i.e., cessation of general movement accompanied by flatline ACC measurements) or loss of connection (i.e., sudden stop in data reception without prior changes in movement or ACC patterns). Each day of post-Eilat spring migration was then classified as either migratory or stopover, with migratory days defined as days in which the individual’s total distance covered > 40 km and/or included a beeline distance between roosting sides of > 19 km (cf. mixed model method from [52]). Next, total distance covered during post-Eilat spring migration was calculated by adding up the total distance covered (from hourly GPS track) per migration day.

Additional information on wintering locations in the following season could be obtained from 13 tagged individuals that survived until the next winter. These data were used to calculate total spring migration distance for these individuals, by adding up the beeline distances (measured as rhumb line) between wintering grounds and Eilat stopover (of the next year) and between Eilat stopover and breeding grounds (of the current/capture year; “post-Eilat” distances to the suspected breeding site). While this measure underestimates the actual length of the migratory tracks and obscures potentially important path deviations taken by the birds and interannual variation, it incorporates the travel through Eilat as a major stopover site, and might better reflect the variation in the distance to the goal area (assuming high breeding-site fidelity; [32, 35, 53]) among adults compared to an estimate of the actual migration path (e.g., the total length of all 1-h or 1-day displacements).

### Demographic and migration statistical analyses

All statistical analyses and visualizations were performed in R version 4.1.2 [54]. We first tested whether there were any differences with age or sex in body condition (Student’s *T*-test) and capture date (non-parametric Scheier-Ray-Hare test) for all captured birds (*n* = 54) as well as for survival (alive vs. dead [binomial]; Pearson’s Chi-squared Test) and post-Eilat migration distance (Two-way ANOVA) for GPS-tagged individuals (thus with reduced sample sizes of *n* = 34 and *n* = 19 respectively). We also tested whether there were differences in body condition between surviving and non-surviving individuals (*n* = 27 for females and *n* = 7 for males) using a Student’s T-test. We considered results with *p* values of < 0.05 as significant.

Additionally, we tested whether steppe buzzard body condition was associated with capture date and/or post-Eilat migration distance (H-2a), and whether these associations were influenced by age. For this we used two sets of linear regression models with either capture date or post-Eilat migration distance as dependent variable and body condition and/or age as explanatory variables. Each set of models contained a null model, a model with only body condition as explanatory variable and two models with both body condition and age either as additive or interacting explanatory variables. After running all models, we used a model selection function (*aictab* from the *AICcmodavg* package [55]) to identify the best fitting model(s) (ΔAICc < 2 [56]). While the set of capture date models was run separately for each sex, the model with post-Eilat migration distance was only ran for females due to insufficient sample size for males (i.e., one juvenile and two adults). The selected models were fitted using the *stats* package [54] and numeric variables were centered and scaled. Lastly, we also tested whether there were correlations between total migration distance and post-Eilat migration distance (*n* = 13) and between capture date and post-Eilat migration distance, using Pearson’s correlation tests (*stats* package).

### Microbiota DNA extraction and sequencing

We used a Qiagen PowerLyzer PowerSoil DNA Kit to extract DNA from our samples. We used a modified version of the kit’s protocol, slightly reducing the amount of Bead Solution (615 µl) and Solution C1 (50 µl) at the beginning of the protocol. These reduced volumes allowed us to transfer all the liquid containing DNA throughout the subsequent parts of the protocol, which we found increased DNA yield. We heated the tubes to 65 °C for 10 min before homogenization as recommended for fecal material (Mobio Laboratories Inc 2018) and then performed bead-beating with a PowerLyzer homogenizer set at 3500 rpm for 16 cycles of 30 s on and 30 s off.

We controlled for potential bacteria introduced through the DNA extraction process [57, 58] by randomly grouping sets of samples for extraction, thereby preventing any bacterial contaminants from confounding potential relationships between bacterial communities and migratory factors of interest. Preliminary DNA extraction tests on cloacal swabs taken from the buzzards outside of this study has poor success when the swab was included in the homogenization tube, probably because the swab did not leave sufficient room for proper homogenization. Therefore, we extracted fecal material from the swab as follows: (1) shake the swab in the ethanol so that fecal material would fall off, (2) remove the swab from its tube and store it for possible re-extraction, (3) spin down the tube that had held the swab to a pellet of fecal material that had fallen off the swab, (4) pipette off the ethanol, (5) pipette in the Bead Solution plus Solution C1 to resuspend the pellet, and (6) pipette the resulting mix into the bead tubes for homogenization. Samples for which the extraction resulted in low amounts of DNA were re-extracted. If re-extraction again yielded low amounts of DNA, the extractions were combined. Combining extractions did not result in any general microbiota differences among the post-filtering samples (see "Microbiota sequence data pre-processing and filtering" and Additional file 1: Results S1).

We used 5 µL of each sample to quantify the DNA with a Qubit fluorometer and then concentrated all samples to 40 µL of volume using a Centrivap vacuum centrifuge. Half of each sample (20 µL) was sent to the Argonne Sequencing Center at Argonne National Laboratory, Lemont, IL, USA where PCR amplification of the V4 region of the 16S rRNA gene was conducted in triplicate for each sample. The PCR primers for the V4 region (515F and 806R) contained adapter sequences for Illumina sequencing and Golay barcodes [59] on the forward primer. Each PCR had 12.5 µL of QuantaBio’s AccuStart II PCR ToughMix, 9.5 µL of MO BIO PCR Water (Certified DNA-Free), 200 pM of each primer, and 1 µL of DNA (more DNA was used if the first PCR failed). The PCR protocol had an initial denature at 94 °C for 3 min, then it cycled 35 times at 94 °C for 45 s, 50 °C for 60 s, and 72 °C for 90 s, and ended with a final hold at 72 °C for 10 min. The triplicate PCRs were combined for each sample and then a pool was made of equimolar amounts of all our buzzard samples (plus additional species for other projects) before sequencing. Sequence data was produced from two runs of an Illumina HiSeq 2500 machine set to yield 150 bp paired-end data.

### Microbiota sequence data pre-processing and filtering

We processed the sequence data in R version 4.1.2 [54]) following the methods detailed in [60]. We trimmed off the first 10 base pairs of each read and then used *DADA2* [61] to infer amplicon sequence variants (ASVs) based on the pool of all sequence reads. We merged the forward and reverse reads and then filtered out chimeric sequences. Taxonomic information was assigned to the sequences using the SILVA 138.1 database [62, 63]. Sequences were aligned using *DECIPHER* [64]. A maximum likelihood phylogeny was inferred using the package *phangorn* [65]. The table of the ASVs was joined with taxonomic information, the phylogeny, and metadata on the buzzards in the package *phyloseq* [66] for analyses of the bacterial communities.

Two negative control samples went through the DNA extraction protocol along with the 54 collected buzzard samples. These two negative control samples, along with an additional blank control sample (Invitrogen UltraPure water), went through 16S rRNA PCR amplification and sequencing. These three control samples were then used to identify any bacterial contaminants that arose from the laboratory environment. We used the prevalence method (0.5 threshold) in the *decontam* package [67], to identify 20 ASV sequences as contaminants, which were then removed from the dataset. Any ASV sequences that were not assigned to the kingdom Bacteria, that were identified as mitochondria or chloroplasts, or which could not be resolved to the phylum level were also removed (*n* = 57), resulting in a final dataset of 1508 bacterial ASVs. Six buzzard samples were removed from the dataset due to problems in PCR amplification (i.e., average post-PCR concentration < 7 ng/µl). The average number of reads per individual for the remaining samples was 48,391 (range = 6825–76,393). After examining rarefaction curves of the data (Additional file 1: Figure S2), we set a minimum read depth of 10,000 reads for our samples, which led to the removal of one additional sample from the dataset. To standardize the sequencing efforts, the remaining samples were then rarefied to the sequencing depth of the sample with the lowest number of reads (14,523 reads) using the *rarefy_even_depth* function from the *phyloseq* package (random seed 999). After rarefaction, the final microbiota dataset included a total of 1322 unique ASVs across 47 individuals. The dataset includes three samples from combined extractions (see "Microbiota DNA extraction and sequencing"), which did not differ from the rest of the samples in general microbiota features (Additional file 1: Results S1) and could thus be left in the dataset.

### Microbiota characterization and comparisons

Microbial α-diversity was measured by the Shannon’s diversity index, Chao1 richness index (*phyloseq* package), and Faith’s phylogenetic diversity (*picante* package [68, 69]). We used log_10_ and inverse transformations for Shannon and Faith’s PD, respectively, to conform to normal distributions. To examine microbial differences between males and females (H-1), we performed an initial examination of general demographic differences in microbial diversity using a two-way ANOVA including both sex and age. Subsequent examinations of links with migration parameters were performed only for females (*n* = 36, and *n* = 30 for survival). We used linear regression models (*stats* package) to test whether there were correlations of the different α-diversity measures with body condition (H-3a) and capture date (H-2b) (both continuous data). Additionally, we determined whether the α-diversity measures differed between survivors and non-survivors (H-3b) (categorical data – excluding individuals that lost GPS connection) by using Student’s *T*-tests.

We then investigated microbial community composition (β-diversity) using distances calculated by four different metrics: Unifrac, Weighted Unifrac, Jaccard and Bray–Curtis (*phyloseq* package). Permutational multivariate analyses of variance (PERMANOVAs) were performed using the *adonis2* function with 9999 permutations (*vegan* package [70]) to test whether the bacterial communities were significantly different (dissimilarity) between sex or age (H-1) and survival (H-3b), and whether bacterial communities varied with body condition (H-3a) and capture date (H-2b). Comparisons of bacterial communities between groups (i.e., sex, age, and survival) additionally included testing for differences in within-group variance (homogeneity of variance) using the *betadisper* function (*vegan* package). Differences in microbial communities were visualized using multidimensional scaling (MDS) analyses. Group dissimilarity (*adonis2*) was tested with sex and age included as additive explanatory factors using *by* = *“margin”* to assess the marginal effects of the tested factors instead of assessing them sequentially. Since body condition, capture date and survival were not found to differ with age and microbiota examinations of these parameters were only carried out for females, there was no need to include any additional demographic factors into the variance and dissimilarity models for these parameters.

To determine specific bacteria taxa leading to community differences, we used an analysis of composition of microbiomes with bias correction (ANCOM-BC, *ANCOMBC* package [71]) to identify which phyla and genera (present in > 10% of samples) showed significant differences in abundance between the demographic groups (H-1), body condition (H-3a), capture date (H-2b), and between survivors and non-survivors (H-3b—excluding individuals that lost GPS connection). We considered taxa to be differentially abundant if the Benjamini–Hochberg adjusted *p* < 0.01 and *W*-statistics above the 85% percentile.

Results on general demographic differences in microbiota composition described below include all sex and age groups. However, due to limited sample size for males, the results on how body condition, arrival time and survival relate to microbiota diversity and composition are only described for females (*n* = 36, survival: *n* = 30). Results on males (*n* = 13, survival: *n* = 6) are included for the sake of completeness in Additional file 1: Results S2*.*

## Results

### Variation in body condition, migration timing and distance and survival in relation to age and sex

Differences in body condition of juvenile and adult individuals were only marginally significant in females (mean SMI ± s.d.; juveniles: 622.5 ± 73.0 g, *n* = 16; adults: 666.6 ± 68.2 g, *n* = 20; Student’s *T*-test: *t* = − 1.851, df = 31.24, *p* = 0.074) and non-significant in males (juveniles: 526.9 ± 51.0 g, *n* = 7; adults: 544.3 ± 60.4 g, *n* = 11; Student’s *T*-test: *t* = − 0.656, df = 14.53, *p* = 0.522). Capture date also did not differ significantly by sex (*p*_adj_ = 0.123) or age (*p*_adj_ = 0.327) (Additional file 1: Table S1). Among the tagged individuals (*n* = 34, not including individuals with tags that lost connection), the overall survival rate was 56%, which did not differ significantly by sex (*p*_adj_ = 0.725) or age (*p*_adj_ = 0.490) (Additional file 1: Table S1). In contrast to our expectation (H-3b), we did not find significant differences in body condition between survivors and non-survivors for both females (surviving: 675.1 ± 69.9 g, *n* = 16; non-surviving: 657.8 ± 64.1 g, *n* = 11; Student’s *T*-test: *t* = − 0.664, df = 22.85, *p* = 0.514) and males (surviving: 618.3 ± 37.3 g, *n* = 3; non-surviving: 570.9 ± 20.1 g, *n* = 4; Student’s *T*-test: *t* = − 1.995, df = 2.87, *p* = 0.144) (Additional file 1: Figure S3). For all individuals that did not survive spring migration (*n* = 15), median distance until death was 0.73 km from the capture location in Eilat (range 0.09–827 km), with most individuals (*n* = 11) dying within 1 km from their capture location. Mean duration until death was 6.7 ± 6.6 days (range 0–23 days). For the surviving individuals (*n* = 19), post-Eilat migration distance was found to differ significantly between juvenile (mean distance ± s.d. units 10^3^ km: 2.31 ± 0.94, *n* = 7) and adult (4.38 ± 1.74, *n* = 12) individuals (*p*_adj_ = 0.018; Additional file 1: Table S1) but differences were only marginally significant between females (3.29 ± 1.61, *n* = 16) and males (males: 5.36 ± 2.01, *n* = 3) (*p*_adj_ = 0.078; Additional file 1: Table S1).

The best fitting model (ΔAICc < 2) explaining female capture date included only body condition as an explanatory variable (Additional file 1: Table S2). In line with our hypothesis (H-2a), this model indicated a negative association between capture date and body condition for females (linear regression: *R*^2^ = 0.17, *F*_1,34_ = 6.77, *p* = 0.014, *n* = 36; Fig. 1d). For males, however, the best fitting model was the null model (Additional file 1: Table S2), indicating there was no relationship between male capture date and body condition (linear regression: *R*^2^ < 0.001, *F*_1,16_ < 0.001, *p* = 0.986, *n* = 18; Fig. 1d). The model selection function performed on female post-Eilat migration distance included three models with ΔAICc values < 2. Since the three best fitting models included the null-model (ΔAICc = 1.708), there is no conclusive support for an association between post-Eilat migration distance and SMI, presumably due to the limited sample size when separated by age (*n* = 10 for adult females and *n* = 3 for juvenile females; Additional file 1: Table S2). However, for all individuals together, post-Eilat migration distance by itself was negatively correlated to capture date (Pearson’s correlation test: *R* = − 0.60, *t* = − 3.077, *df* = 14, *p* = 0.007, *n* = 19; Fig. 1b,e), indicating earlier arriving individuals generally have a longer post-Eilat migration distance. Additionally, post-Eilat migration distance was strongly positively correlated with total migration distance (Pearson’s correlation test: *R* = 0.79, *t* = 4.254, *df* = 11, *p* = 0.001, *n* = 13; Fig. 1b,c,f).

### Microbiota: general description and demographic differences

**Fig. 2:**
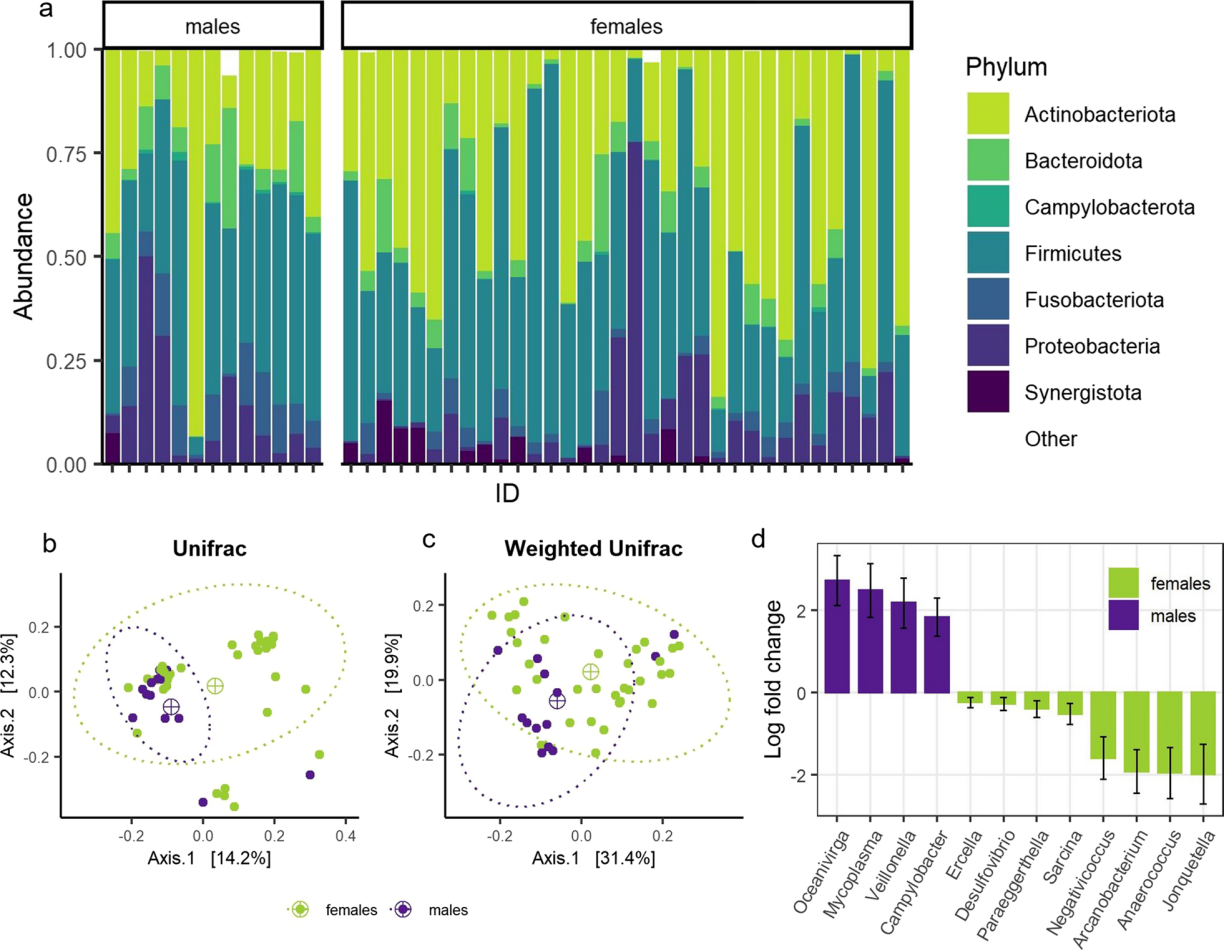
Comparison of male and female steppe buzzard microbiota composition. **a** Relative abundance of the most common phyla (relative abundance of > 1%) per individual, **b** MDS (multidimensional scaling) plots of unweighted Unifrac distances and **c** Weighted Unifrac distances colored by sex (males in dark green, females in light green), including ellipses (dashed lines) of 95% confidence around centroids (⊕). **d** Significantly differentially abundant genera as determined by ANCOM-BC analysis, with positive log-fold change values indicating relatively higher abundances in males (purple) and negative log-fold change values indicating higher relative abundances in females (green)

Across all samples (*n* = 47), the most abundant phyla were Firmicutes (mean relative abundance ± s.d.: 41.6 ± 19.0%), Actinobacteriota (35.1 ± 23.8%), Proteobacteria (10.6 ± 14.3%), Bacteriodota (5.9 ± 6.2%), Fusobacteriota (4.6 ± 4.5%) and Synergistota (1.7 ± 3.3%) (Fig. 2a). The most abundant genera were *Corynebacterium* (relative abundance: 21.5 ± 22.8%: prevalence: 100%), *Varibaculum* (9.8 ± 8.0%: 100%), *Clostridium* sensu stricto 1 (9.2 ± 12.8%: 100%) and *Escherichia-Shigella* (7.3 ± 13.1%: 100%). All other genera with prevalence of > 80% are shown in Additional file 1: Table S3. Overall, the average prevalence of ASVs was 13.9%. A total of 16 ASVs occurred in > 90% of the samples, however most (*n* = 11) at low (< 1%) relative abundances (Additional file 1: Table S4).

We did not find any significant differences in microbial α-diversity by sex (Shannon: *F*_1,44_ = 0.413; *p* = 0.524, Chao1: *F*_1,44_ = 0.108, *p* = 0.744; Faith’s PD: *F*_1,44_ = 1.507, *p* = 0.226; Additional file 1: Table S5) or age (Shannon: *F*_1,44_ = 1.289, *p* = 0.262, Chao1: *F*_1,44_ = 0.000, *p* = 0.993; Faith’s PD: *F*_1,44_ = 0.001, *p* = 0.970; Additional file 1: Table S5). However, in accordance with our hypothesis (H-1), the community compositions (*β*-diversity) were found to differ significantly between females and males (unweighted Unifrac: *p* = 0.006, Fig. 2b; Weighted Unifrac: *p* = 0.011, Fig. 2c; Jaccard: *p* = 0.010, Additional file 1: Figure S4a; Bray–Curtis: *p* = 0.011, Additional file 1: Figure S4b; Table S5), without differences in dispersion of their communities (*p* > 0.05 for all metrics; Additional file 1: Table S5). Differential abundance analyses revealed higher abundances of the genera *Oceanivirga*, *Mycoplasma*, *Veillonella* and C*ampylobacter*, and lower abundances of *Ercella*, *Desulfovibrio*, *Paraeggerthella*, *Sarcina*, *Negativicoccus*, *Arcanobacterium* and *Jonquetella* in males (Fig. 2d; Additional file 1: Table S5). We found that community differences with age were not significant (communities and dispersal *p* > 0.05 for all metrics; Additional file 1: Table S5). However, we found significantly higher abundances in the genera *Arcanobacterium*, *Negativicoccus* and *Salmonella* in adult individuals (Additional file 1: Table S5).

### Microbiota associations with body condition

Female (*n* = 36) body condition (SMI) upon capture was positively correlated with the Shannon *α*-diversity index (*R*^2^ = 0.22, *F*_2,31_ = 4.264, *p* = 0.023) but not with the Chao1 index (*R*^2^ = 0.03, *F*_2,31_ = 0.506, *p* = 0.608) or Faith’s PD (*R*^2^ = 0.06, *F*_2,31_ = 1.017, *p* = 0.373). We did not find a significant relationship between body condition and composition of the microbiota community (unweighted Unifrac: *R*^2^ = 0.04, *F*_1,32_ = 1.270, *p* = 0.148; Weighted Unifrac: *R*^2^ = 0.04, *F*_1,32_ = 1.450, *p* = 0.185; Jaccard: *R*^2^ = 0.04, *F*_1,32_ = 1.328, *p* = 0.123; Bray–Curtis: *R*^2^ = 0.05, *F*_1,32_ = 1.534, *p* = 0.110), but did find that the relative abundance of the genus *Escherichia-Shigella* significantly increased with decreasing body condition (ANCOM-BC: *W* = − 5.437, *q* < 0.001). These results partly support our third hypothesis stating that microbiota would differ with body condition (H-3a).

### Microbiota associations with capture date

**Fig. 3:**
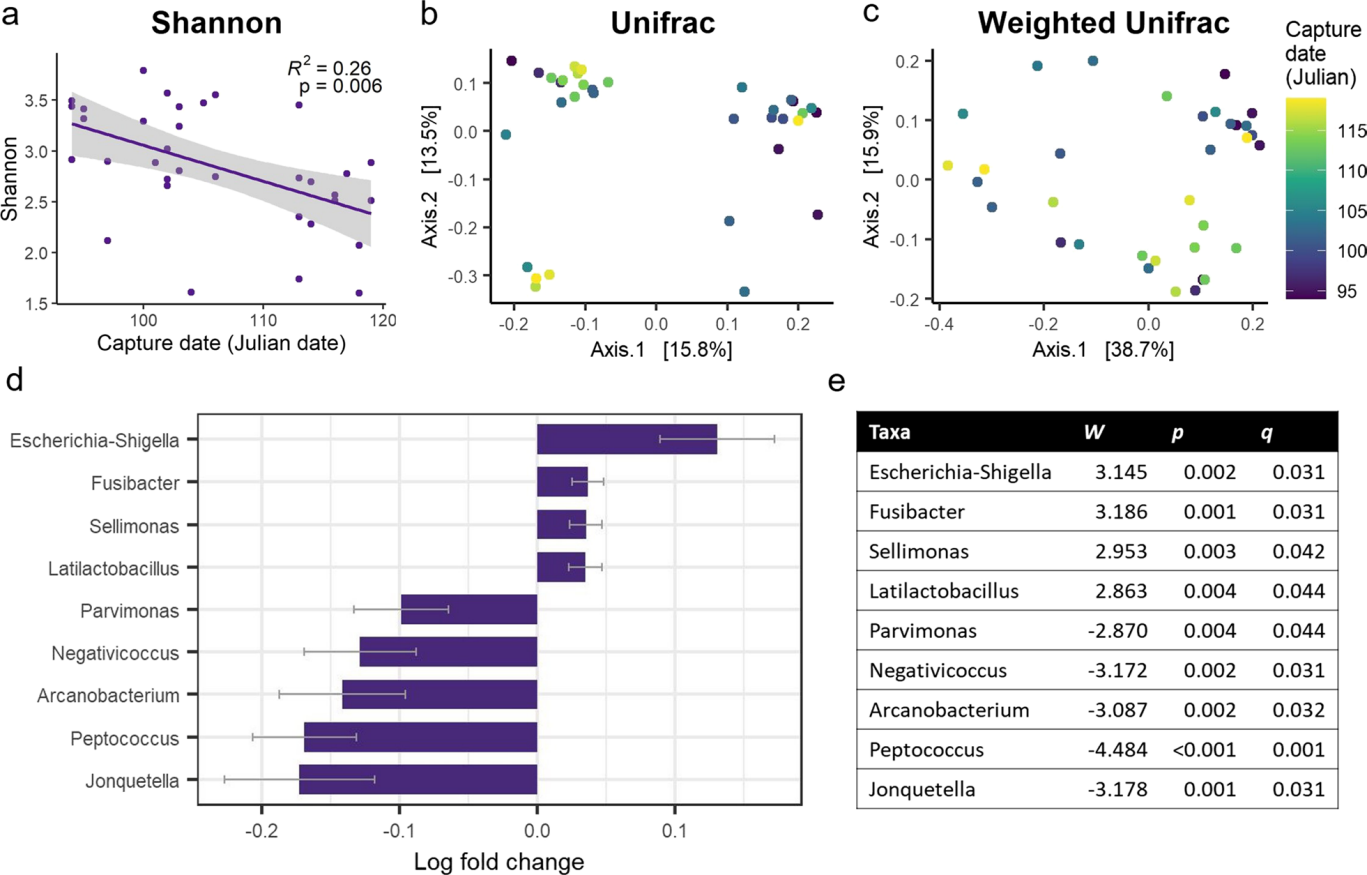
Associations between capture date and microbiota measures in female steppe buzzards. **a** Capture date was significantly negatively related to Shannon diversity. **b** MDS (multidimensional scaling) plots of unweighted Unifrac distances and **c** weighted Unifrac distances colored by capture date, ranging from purple (earlier arrival) to yellow (later arrival). **d** Significant differentially abundant genera as determined by ANCOM-BC analysis, with positive log-fold change values indicating the taxa increased with later arrival date and negative log-fold change values indicate a that the taxa decreased with later arrival date, and **e** corresponding *W-*, *p-* and *q-*values

In accordance with our second hypothesis (H-2b), we found that female (*n* = 36) capture date was significantly negatively correlated with Shannon *α*-diversity (*R*^2^ = 0.28, *F*_2,31_ = 6.062, *p* = 0.006; Fig. 3a), but not with Chao1 index (*R*^2^ = 0.01, *F*_2,31_ = 0.083, *p* = 0.921) or Faith’s PD (inv. transf.; *R*^2^ = 0.02, *F*_2,31_ = 0.364, *p* = 0.698). Moreover, capture date was also significantly correlated with community composition using the unweighted Unifrac metric (*R*^2^ = 0.05, *F*_1,32_ = 1.535, *p* = 0.048; Fig. 3b), Jaccard metric (*R*^2^ = 0.06, *F*_1,32_ = 1.927, *p* = 0.015; Additional file 1: Figure S5a), and Bray–Curtis metric (*R*^2^ = 0.07, *F*_1,32_ = 2.431, *p* = 0.017; Additional file 1: Figure S5b), but the correlation was only marginally significant for the Weighted Unifrac metric (*R*^2^ = 0.06, *F*_1,32_ = 2.003, *p* = 0.067; Fig. 3c). Relative abundance of the phylum Proteobacteria (ANCOM-BC: *W* = 2.813, *q* = 0.027) increased with capture date, while abundance of the phylum Synergistota (ANCOM-BC: *W* = − 2.907, *q* = 0.027) decreased with capture date. Additionally, abundances of the genera *Latilactobacillus*, *Sellimonas*, *Fusibacter* and *Escherichia*-*Shigella* increased with capture date, while abundances of the genera *Jonquetella*, *Peptococcus*, *Arcanobacterium*, *Negativicoccus* and *Parvimonas* decreased with capture date (Fig. 3d,e), which additionally supports our hypothesis that arrival time is connected to differences in gut microbiome (H-2b).

### Microbiota associations with survival

**Fig. 4:**
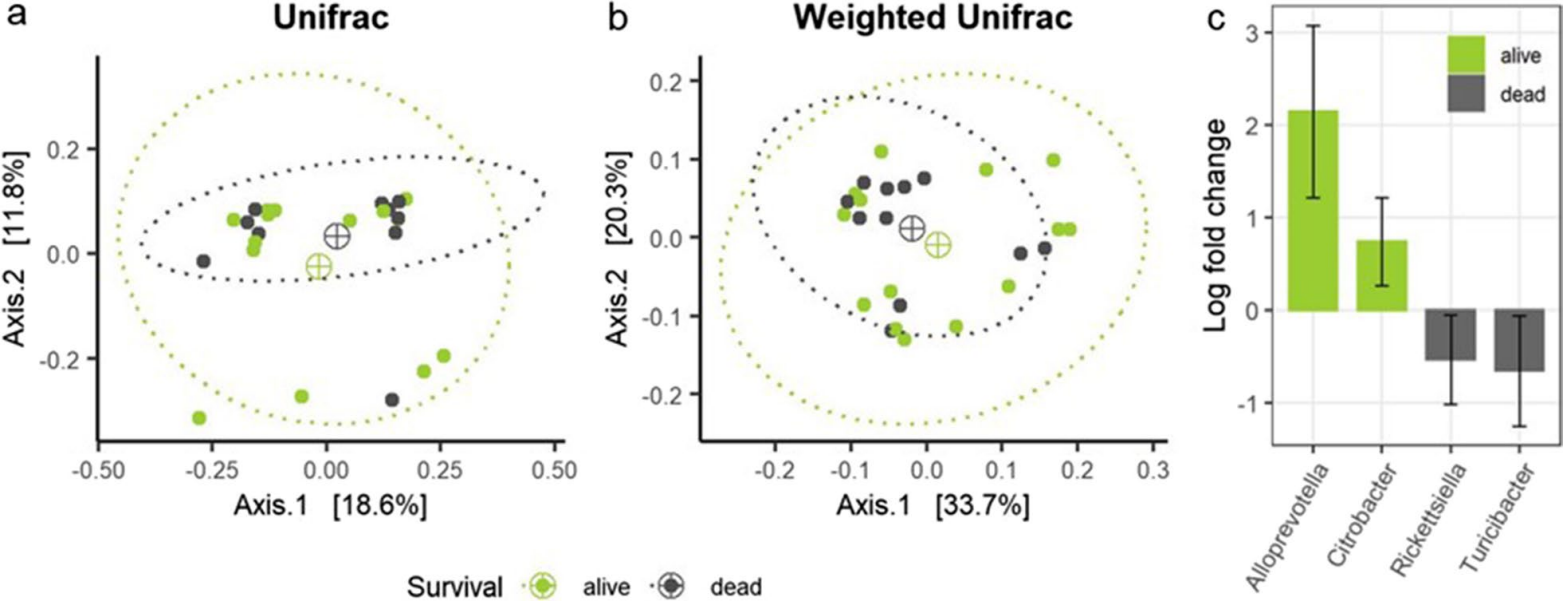
Associations between spring migration survival and microbiota measures in female steppe buzzards. **a** MDS (multidimensional scaling) plots of unweighted Unifrac distances and **b** weighted Unifrac distances colored by female survival (alive in green, dead in purple), including ellipses (dashed lines) of 95% confidence around centroids (⊕). **c** Significant differentially abundant genera as determined by ANCOM-BC analysis, with positive log-fold change values indicating higher relative abundance in surviving individuals (“alive”, green) and negative log-fold change values indicating higher relative abundance in non-surviving in individuals (“dead”, grey)

Since we found no difference in overall spring-migration survival rate between juveniles and adults (Section *Variation in body condition, migration timing and distance in relation to age and* sex), the two age classes were grouped together for subsequent microbiota analyses. Contrary to our expectations (H-3b), female individuals that did (*n* = 14) and did not (*n* = 11) survive post-Eilat spring migration did not differ in *α*-diversity (*p* > 0.05 for all metrics; Additional file 1: Table S6) or community composition (*β*-diversity: *p* > 0.05 for all metrics; Fig. 4a,b; Additional file 1: Figure S6; Table S6) or dispersal (*p* > 0.05 for all metrics; Additional file 1: Table S6). However, surviving individuals had significantly higher relative abundances of the genera *Alloprevotella* and *Citrobacter* (ANCOM-BC: *W* = − 2.306, *q* < 0.001 and *W* = − 1.565, *q* < 0.001, respectively; Fig. 4c), whereas non-surviving individuals had significantly higher abundances of the genera *Rickettsiella* and *Turicibaceter* (ANCOM-BC: *W* = 1.099, *q* < 0.001 and *W* = 1.090, *q* < 0.001, respectively; Fig. 4c). We also tested associations between the microbiota and these same parameters when considering survival within 5- and 10-days post trapping rather than overall migratory survival; results were consistent across all three time periods and are therefore not shown.

## Discussion

In this study, we investigated links between cloacal bacterial microbiota composition, body condition, and migratory patterns in GPS-tagged birds. We found that steppe buzzards that arrived earlier to the stopover in Eilat generally had better body condition and longer subsequent migratory journeys. This relationship did not differ with sex or age, despite juveniles having a generally shorter migration journey than adults. In contrast, microbiota composition (*β*-diversity) was markedly different between males and females. Subsequent investigation of just female buzzards revealed that body condition was positively correlated with microbial diversity (Shannon index) and negatively correlated with abundance of the genus *Escherichia-Shigella*, whereas capture date was negatively correlated with microbial diversity and positively correlated with abundance of the genus *Escherichia-Shigella*. Additionally, earlier arriving individuals had a distinct bacterial community composition, characterized by higher prevalence and abundance of specific bacterial genera, including *Jonquetella* and *Peptococcus*. In contrast to our expectations, survival was not associated with inter-individual variation in body condition, microbiota diversity or composition.

### Migration and survival of steppe buzzards

Birds arriving to Eilat during spring migration have just finished crossing a large ecological barrier; the ca. 1800 km Sahara Desert. Individuals crossing such ecological barriers must fly for longer periods of time, expending more energy while experiencing limited abilities to feed and replenish energy along the way, which may result in deteriorated body condition upon arrival at their next stopover [72, 73]. Specifically, crossing of the Sahara Desert during spring migration has been connected to elevated levels of mortality in other raptor species, such as osprey (*Pandion haliaetus*), marsh harrier (*Circus aeruginosus*), and Montagu’s harrier (*Circus pygargus*) [74]. While high mortality rates are also a common phenomenon for steppe buzzards passing through Eilat in spring (pers. comm. IBRCE, Eilat Birding Center), the overall mortality of 46% of tagged individuals found in this study is likely additionally increased due to a potential capture- and tagging bias towards individuals with lower body condition [33]. We only captured individuals attracted to a baited trap, which has been shown to indicate high refueling motivation in steppe buzzards [75]. At the same time, individuals with relatively higher body conditions and energy availability may have been strong enough to continue migrating after crossing the ecological barriers and may have stopped at more northern locations instead of at the first suitable stopover locations around Eilat. However, despite the potential bias towards lower body conditions, earlier arriving individuals generally had better body condition at capture than later arriving individuals. This trend has previously been found for steppe buzzards measured in Eilat’s stopover and could indicate higher migratory performance of early arriving individuals that generally migrate more efficiently or encounter more favorable environmental conditions and so experience less body condition decline along their migratory route [33]. An alternative explanation is the variation in the anticipated goal area among adult birds, assuming high breeding- and wintering-site fidelity as reported for the closely related common buzzard (*Buteo buteo buteo* [53]) and many other raptor species [32, 35], and supported by data from three of our tracked birds with data from > 2 years (Additional file 1: Figure S8). The significant negative correlation of arrival date with both body condition and post-Eilat migration distance suggests that individuals might aim to arrive at a stopover earlier and in better condition relative to the remaining post-stopover distance. Therefore, the better body condition of early arriving adults could reflect not only variation among individuals in the past events prior to arrival to Eilat, but also in the adjustments they made in relation to the subsequent migration towards the goal area.

### Microbiota characterization of steppe buzzards in a major migration bottleneck

Steppe buzzard microbiota was dominated by the same phyla found in most bird species; Firmicutes, Actinobacteriota, Proteobacteriota and Bacteriodota [76]. We additionally found relatively high abundances of Fusobacteriota, a phylum that seems to be a common feature in the microbiota of carnivorous birds [77], and the genus *Corynebacterium* (most prevalent and abundant genus in our study), abundances of which have previously found to be elevated in migratory birds [16, 17, 19].

The microbiota composition (*β*-diversity) of male and female individuals differed significantly. While microbiota diversity (*α*-diversity) was similar, there were 12 differentially abundant genera—with higher abundances of the genera *Oceanivirga*, *Mycoplasma*, *Veillonella* and *Campylobacter* in males, and higher abundances of the genera *Negativicoccus*, *Arcanobacterium*, *Anaerococcus* and *Jonquettella* in females. Differences between the microbiota composition of sexes in human and animal studies is not uncommon (as reviewed in [78]). Bird studies have also found microbiota differences with sex [14, 27, 28] though not consistently (e.g., [17]). The presence of such differences in the microbiota generally seems to be associated with other sex-related differences like hormone levels [27], diet [28] or differential changes in body size or weight [79]. Sampled female steppe buzzards were roughly 20% larger and heavier than their male counterparts, which by itself could potentially drive microbiota differences [80]. In addition, females might experience different time versus energy (body condition) pressures during spring migration due to the need to (immediately) commence breeding upon reaching the breeding grounds [32]. Maintaining superior body and energetic conditions throughout migration might additionally come with differences in refueling, foraging patterns, and other behaviors, which could explain the differences we observed between male and female steppe buzzards. However, other factors known to cause sexual dimorphism in males and females and are known to influence microbiome composition, including immune function [81] and hormone levels [27].

### Migration-microbiota links in steppe buzzards

Our results confirm that the microbiota composition of migrating female steppe buzzards is linked to variation in migratory patterns. Certain features of the steppe buzzard microbiota composition that changed with arrival date, for example the negative correlation with *α*-diversity and relative abundance of the genera *Escherichia-Shigella*, could be mainly caused by its interaction with body condition. Not only are arrival date and body condition negatively correlated, body condition itself was also positively correlated with *α*-diversity and negatively correlated to *Escherichia-Shigella*. Microbial *α*-diversity can change with host health and condition because decreases in *α*-diversity have been connected to disease and death [10, 25]. Moreover, decreased *α*-diversity can make the host more vulnerable to invasion by opportunistic and pathogenic taxa [25, 82]. Similar processes could potentially have affected the steppe buzzard microbiota, with lowered body condition being related to both lower *α*-diversity and increased abundances of the potentially pathogenic members of the genera *Escherichia-Shigella* [83]. The higher abundances of the genera *Jonquetella*, *Peptococcus, Arcanobacterium,* and *Negativicoccus* may be more directly connected to exposures to different environments along migration routes or the distance travelled. *Peptococcus*, for example, includes anaerobic organisms that are related to respiratory glucose metabolism [84, 85], which might play a beneficial role during longer migratory journeys. Steppe buzzards are also known to significantly increase body mass prior to starting migration and require refueling along the way to be able to fly back to the breeding grounds [33]. The identified taxa could thus also be a signature of certain pre-migratory (on wintering grounds) or recent pre-stopover environments/refueling patterns associated with longer migration distances, and/or reflect the level of energetic stress experienced while crossing the Sahara Desert. However, this study only provides a “snapshot” in time of the microbiota communities, and without sampling at multiple time points, shotgun sequencing to assess gene content, or metabolomics of the collected samples, assigning specific functions or causes for the presence of the identified bacterial lineages can only be speculative.

Contrary to our hypothesis, we did not find any significant differences in body condition, microbiota diversity or composition between females that survived and did not survive spring migration. While previous studies have found a connection between microbiota and survival, the results actually vary significantly between studies and species, with, for example, higher α-diversity for non-surviving blue tits (*Cyanistes caeruleus, n* = 54 [23]), higher β-diversity for non-surviving barn swallows (*Hirunda rustica, n* = 42 [24]), and higher abundances of potentially pathogenic genera in non-surviving Seychelles warblers (*Acrocephalus sechellensis*, *n* = 268 [26]). While this could mean that microbiota signatures of survival are species- and location dependent, survival in these studies was determined by whether a sampled bird was recaptured [24] or resighted [23, 26] in the same area after several months and up to a year post-capture. Depending on the species, such methods tend to overestimate death rates because they do not differentiate among dispersal events, incomplete sampling, and true death, and are not comparable with GPS-ACC measurements that can record incidents of these events for all tracked individuals in a direct and detailed manner [86]. We further emphasize that all the three studies cited above either focused on resident non-migratory populations (tits and warblers), or on a migratory species that was sampled only in the breeding site in successive years (swallows). We did not find any previous study that examined the relationship between host survival and microbiota diversity or composition *during* migration, and our lack of support of H-3b should not discourage further efforts for examining these links during challenging migration journeys, where and when variation in microbiota might still affect the fate of individuals.

The absence of a strong link between microbiota and directly monitored survival in this study is accompanied by an unexpected absence of differences in body condition. This could suggest that the sampled individuals all showed signs of physical stress due to recently crossing the Sahara Desert, which, as mentioned above, is common in migratory birds crossing ecological barriers [33, 72, 73]. These similar levels of physical stress and exhaustion thus might have been reflected similarly in their microbiota composition at the time of capture, while survival was more heavily determined by post-capture events. Since the mortality rate of 46% found in this study is elevated compared to other studies and most of these individuals have been found dead within 1 km from their capture location, it is possible that other explanations are involved. For example, is possible that the capture, sampling, and attachment of GPS-tags caused additional stress to already physically stressed and exhausted birds. Capture and restraint of birds are known to increase corticosterone levels [87, 88] whereas carrying a harness and additional weight from a GPS-tag have been shown to influence bird behavior and survival (e.g., [89, 90]). Even though we used recommended handling and harnessing techniques and stayed below the recommended threshold of 5% [49], it is possible that these processes had unforeseen adverse effects on the steppe buzzard. This could have caused more individuals to die than one would expect based on physical condition upon capture alone, especially during a time of high physical stress. Other, non-physical, causes of mortality that are not reflected in microbiome composition, like predation, hunting or other human-induced effects (e.g. collisions with powerlines or poisoning) [74, 91, 92], can be excluded for most of the deceased birds. We were able to locate the carcasses of all birds (*n* = 11) that died close to their capture location (i.e., within the date plantations) within two days, none of which showed any signs for predation, hunting or other potentially deadly injuries upon external examination.

## Conclusions and future directions

This study provides new evidence that variation in microbiota of a migratory bird can be linked to variation in body condition during migration and migratory patterns. Our key finding—the higher *α*-diversity and distinct microbiota composition found in early arriving birds in relation to their body condition and migratory distance/route—could not have been obtained without the movement data from the GPS-tracked individuals. Further work is needed to develop a more detailed understanding of the relationship between bacterial diversity and migration, and to identify bacterial groups associated with certain migratory characteristics, such as areas visited or (recent) refueling sessions. Subsequent research should aim to collect more frequent and detailed information on migration routes (e.g., number and length of visited stopovers) and performance (e.g., speeds, flapping rate, and energy expenditure), by measuring and re-sampling tracked individuals on multiple occasions along their migration routes.

## Supplementary Information

**Additional file 1.** Supplementary results, tables, and figures.

## Data Availability

The 16S sequence data associated with this study will be deposited in the Sequence Read Archive under BioProject ID: PRJNA578383 upon acceptance of the manuscript and will then be found at the following link: https://www.ncbi.nlm.nih.gov/sra/PRJNA578383. Movement data will be deposited as open access in Movebank upon acceptance for publication.

## References

[1] Buehler DM, Piersma T (2008). Travelling on a budget: predictions and ecological evidence for bottlenecks in the annual cycle of long-distance migrants. Philos Trans R Soc Lond B Biol Sci.

[2] Tøttrup AP, Klaassen RHG, Strandberg R, Thorup K, Kristensen MW, Jørgensen PS (2012). The annual cycle of a trans-equatorial Eurasian-African passerine migrant: different spatio-temporal strategies for autumn and spring migration. Proc R Soc B Biol Sci.

[3] Webster MS, Marra PP, Haig SM, Bensch S, Holmes RT (2002). Links between worlds: unraveling migratory connectivity. Trends Ecol Evol.

[4] DeSimone JG, Ramirez MG, Elowe CR, Griego MS, Breuner CW, Gerson AR (2020). Developing a stopover-CORT hypothesis: corticosterone predicts body composition and refueling rate in gray catbirds during migratory stopover. Horm Behav.

[5] Battley PF, Piersma T, Dietz MW, Tang S, Dekinga A, Hulsman K (2000). Empirical evidence for differential organ reductions during trans–oceanic bird flight. Proc R Soc B Biol Sci.

[6] Eikenaar C, Hegemann A, Packmor F, Kleudgen I, Isaksson C (2020). Not just fuel: energy stores are correlated with immune function and oxidative damage in a long-distance migrant. Curr Zool.

[7] Lindsay EC, Metcalfe NB, Llewellyn MS (2020). The potential role of the gut microbiota in shaping host energetics and metabolic rate. J Anim Ecol.

[8] Kamada N, Seo S-U, Chen GY, Núñez G (2013). Role of the gut microbiota in immunity and inflammatory disease. Nat Rev Immunol.

[9] Buffie CG, Pamer EG (2013). Microbiota-mediated colonization resistance against intestinal pathogens. Nat Rev Immunol.

[10] Hird SM, Ganz H, Eisen JA, Boyce WM (2018). The cloacal microbiome of five wild duck species varies by species and influenza A virus infection status. mSphere.

[11] Escallón C, Becker MH, Walke JB, Jensen RV, Cormier G, Belden LK (2017). Testosterone levels are positively correlated with cloacal bacterial diversity and the relative abundance of Chlamydiae in breeding male rufous-collared sparrows. Funct Ecol.

[12] Pekarsky S, Corl A, Turjeman S, Kamath PL, Getz WM, Bowie RCK (2021). Drivers of change and stability in the gut microbiota of an omnivorous avian migrant exposed to artificial food supplementation. Mol Ecol.

[13] Phillips JN, Berlow M, Derryberry EP (2018). The effects of landscape urbanization on the gut microbiome: an exploration into the gut of urban and rural white-crowned sparrows. Front Ecol Evol.

[14] Corl A, Charter M, Rozman G, Toledo S, Turjeman S, Kamath PL (2020). Movement ecology and sex are linked to barn owl microbial community composition. Mol Ecol.

[15] Lewis WB, Moore FR, Wang S (2017). Changes in gut microbiota of migratory passerines during stopover after crossing an ecological barrier. Auk.

[16] Risely A, Waite DW, Ujvari B, Hoye BJ, Klaassen M (2018). Active migration is associated with specific and consistent changes to gut microbiota in *Calidris* shorebirds. J Anim Ecol.

[17] Turjeman S, Corl A, Wolfenden A, Tsalyuk M, Lublin A, Choi O (2020). Migration, pathogens and the avian microbiome: a comparative study in sympatric migrants and residents. Mol Ecol.

[18] Skeen HR, Cooper NW, Hackett SJ, Bates JM, Marra PP (2021). Repeated sampling of individuals reveals impact of tropical and temperate habitats on microbiota of a migratory bird. Mol Ecol.

[19] Risely A, Waite D, Ujvari B, Klaassen M, Hoye B (2017). Gut microbiota of a long-distance migrant demonstrates resistance against environmental microbe incursions. Mol Ecol.

[20] Lewis WB, Moore FR, Wang S (2016). Characterization of the gut microbiota of migratory passerines during stopover along the northern coast of the Gulf of Mexico. J Avian Biol.

[21] Noguera JC, Aira M, Pérez-Losada M, Domínguez J, Velando A (2018). Glucocorticoids modulate gastrointestinal microbiome in a wild bird. R Soc Open Sci.

[22] Zaneveld JR, McMinds R, Vega TR (2017). Stress and stability: applying the Anna Karenina principle to animal microbiomes. Nat Microbiol.

[23] Benskin CMcWH, Rhodes G, Pickup RW, Mainwaring MC, Wilson K, Hartley IR (2015). Life history correlates of fecal bacterial species richness in a wild population of the blue tit *Cyanistes caeruleus*. Ecol Evol.

[24] Ambrosini R, Corti M, Franzetti A, Caprioli M, Rubolini D, Motta VM (2019). Cloacal microbiomes and ecology of individual barn swallows. FEMS Microbiol Ecol.

[25] Videvall E, Song SJ, Bensch HM, Strandh M, Engelbrecht A, Serfontein N (2020). Early-life gut dysbiosis linked to juvenile mortality in ostriches. Microbiome.

[26] Worsley SF, Davies CS, Mannarelli M-E, Hutchings MI, Komdeur J, Burke T (2021). Gut microbiome composition, not alpha diversity, is associated with survival in a natural vertebrate population. Anim Microbiome.

[27] Escallón C, Belden LK, Moore IT (2019). The cloacal microbiome changes with the breeding season in a wild bird. Integr Org Biol.

[28] Liu G, Meng D, Gong M, Li H, Wen W, Wang Y (2020). Effects of sex and diet on gut microbiota of farmland-dependent wintering birds. Front Microbiol.

[29] Mendelsohn J (1986). Recoveries and Palaearctic origins of steppe buzzards ringed in South Africa. Safring News.

[30] Israeli Bird Ringing Center (IBRC). Israel Ornithological Center. Society for the Protection of Nature in Israel. www.birds.org.il.

[31] Spina F, Baillie S, Bairlein F, Fiedler W, Thorup K. The Eurasian African Bird Migration Atlas. 2022. https://migrationatlas.org.

[32] Newton I (2008). The migration ecology of Birds.

[33] Gorney E, Yom-Tov Y (1994). Fat, hydration condition, and moult of steppe buzzards *Buteo buteo vulpinus* on spring migration. Ibis.

[34] Shirihai H, Dovrat E, Christie DA, Harris A (1996). The birds of Israel.

[35] Newton I. Population Ecology of Raptors. Berkhamstead UK: Poyser; 1979.

[36] Teyssier A, Lens L, Matthysen E, White J (2018). Dynamics of gut microbiota diversity during the early development of an avian host: evidence from a cross-foster experiment. Front Microbiol.

[37] Izhaki I, Maitav A (2008). Blackcaps *Sylvia atricapilla* stopping over at the desert edge; inter- and intra-sexual differences in spring and autumn migration. Ibis.

[38] Maggini I, Spina F, Voigt CC, Ferri A, Bairlein F (2013). Differential migration and body condition in northern wheatears (*Oenanthe oenanthe*) at a Mediterranean spring stopover site. J Ornithol.

[39] Baker AJ, González PM, Piersma T, Niles LJ, de Lima Serrano do Nascimento I, Atkinson PW (2004). Rapid population decline in red knots: fitness consequences of decreased refuelling rates and late arrival in Delaware Bay. Proc R Soc B Biol Sci.

[40] Morrison RIG, Davidson NC, Wilson JR (2007). Survival of the fattest: body stores on migration and survival in red knots *Calidris canutus islandica*. J Avian Biol.

[41] Duijns S, Niles LJ, Dey A, Aubry Y, Friis C, Koch S (2017). Body condition explains migratory performance of a long-distance migrant. Proc R Soc B Biol Sci.

[42] Anderson AM, Duijns S, Smith PA, Friis C, Nol E (2019). Migration distance and body condition influence shorebird migration strategies and stopover decisions during southbound migration. Front Ecol Evol.

[43] Boshoff AF, Harrison JA, Allan DG, Underhill LG, Herremans M, Tree AJ, Parker V (1997). Steppe Buzzard. The Atlas of Southern African birds.

[44] BirdLife International. Species factsheet: *Buteo buteo*. 2022. http://www.birdlife.org.

[45] Berger D, Mueller H (1959). The bal-chatri: a trap for the birds of prey. Bird-Banding.

[46] du Feu C, Clark J, Fiedler W, Baillie S. EURING exchange code 2000+. Radolfzell, Germany; 2010.

[47] Forsman D (2016). Flight identification of raptors of Europe.

[48] Suh A, Kriegs JO, Brosius J, Schmitz J (2011). Retroposon insertions and the chronology of avian sex chromosome evolution. Mol Biol Evol.

[49] Kenward RE (2001). A manual for wildlife radio tagging.

[50] Fridolfsson A-K, Ellegren H (1999). A simple and universal method for molecular sexing of non-ratite birds. J Avian Biol.

[51] Peig J, Green AJ (2009). New perspectives for estimating body condition from mass/length data: the scaled mass index as an alternative method. Oikos.

[52] Klaassen RHG, Strandberg R, Hake M, Olofsson P, Tøttrup AP, Alerstam T (2010). Loop migration in adult marsh harriers *Circus aeruginosus,* as revealed by satellite telemetry. J Avian Biol.

[53] Martín B, Onrubia A, Ferrer M (2014). Effects of climate change on the migratory behavior of the common buzzard *Buteo buteo*. Clim Res.

[54] R Core Team (2018). R: a language and environment for statistical computing.

[55] Mazerolle MJ. AICcmodavg: model selection and multimodel inference based on (Q)AIC(c). R package version 2.3-1; 2020. https://cran.r-project.org/package=AICcmodavg.

[56] Burnham KP, Anderson DR (2004). Model selection and multimodel inference: a practical information-theoretic approach.

[57] Salter SJ, Cox MJ, Turek EM, Calus ST, Cookson WO, Moffatt MF (2014). Reagent and laboratory contamination can critically impact sequence-based microbiome analyses. BMC Biol.

[58] Weiss S, Amir A, Hyde ER, Metcalf JL, Song SJ, Knight R (2014). Tracking down the sources of experimental contamination in microbiome studies. Genome Biol.

[59] Caporaso JG, Lauber CL, Walters WA, Berg-Lyons D, Huntley J, Fierer N (2012). Ultra-high-throughput microbial community analysis on the Illumina HiSeq and MiSeq platforms. ISME J.

[60] Callahan BJ, Sankaran K, Fukuyama JA, McMurdie PJ, Holmes SP (2016). Bioconductor workflow for microbiome data analysis: from raw reads to community analyses. F1000Res.

[61] Callahan BJ, McMurdie PJ, Rosen MJ, Han AW, Johnson AJA, Holmes SP (2016). DADA2: high-resolution sample inference from Illumina amplicon data. Nat Methods.

[62] Quast C, Pruesse E, Yilmaz P, Gerken J, Schweer T, Yarza P (2012). The SILVA ribosomal RNA gene database project: improved data processing and web-based tools. Nucleic Acids Res.

[63] Glöckner FO, Yilmaz P, Quast C, Gerken J, Beccati A, Ciuprina A (2017). 25 years of serving the community with ribosomal RNA gene reference databases and tools. J Biotechnol.

[64] Wright ES (2015). DECIPHER: harnessing local sequence context to improve protein multiple sequence alignment. BMC Bioinform.

[65] Schliep KP (2011). Phangorn: phylogenetic analysis in R. Bioinformatics.

[66] McMurdie PJ, Holmes S (2013). Phyloseq: an R package for reproducible interactive analysis and graphics of microbiome census data. PLoS ONE.

[67] Davis NM, Proctor DM, Holmes SP, Relman DA, Callahan BJ (2018). Simple statistical identification and removal of contaminant sequences in marker-gene and metagenomics data. Microbiome.

[68] Faith DP (1992). Conservation evaluation and phylogenetic diversity. Biol Conserv.

[69] Kembel SW, Cowan PD, Helmus MR, Cornwell WK, Morlon H, Ackerly DD (2010). Picante: R tools for integrating phylogenies and ecology. Bioinformatics.

[70] Oksanen J, Blanchet GF, Friendly M, Kindt R, Legendre P, McGlinn D, et al. Vegan: community ecology package. R package version 2.5-7; 2020. https://cran.r-project.org/package=vegan.

[71] Lin H, Peddada SD (2020). Analysis of compositions of microbiomes with bias correction. Nat Commun.

[72] Vansteelant WMG, Bouten W, Klaassen RHG, Koks BJ, Schlaich AE, van Diermen J (2015). Regional and seasonal flight speeds of soaring migrants and the role of weather conditions at hourly and daily scales. J Avian Biol.

[73] Efrat R, Hatzofe O, Nathan R (2019). Landscape-dependent time versus energy optimizations in pelicans migrating through a large ecological barrier. Funct Ecol.

[74] Klaassen RHG, Hake M, Strandberg R, Koks BJ, Trierweiler C, Exo K-M (2014). When and where does mortality occur in migratory birds? Direct evidence from long-term satellite tracking of raptors. Hays G, editor. J Anim Ecol.

[75] Perlman Y, Tsurim I (2008). Daring, risk assessment and body condition interactions in steppe buzzards *Buteo buteo vulpinus*. J Avian Biol.

[76] Grond K, Sandercock BK, Jumpponen A, Zeglin LH (2018). The avian gut microbiota: community, physiology and function in wild birds. J Avian Biol.

[77] Waite DW, Taylor MW (2015). Exploring the avian gut microbiota: current trends and future directions. Front Microbiol.

[78] Kim YS, Unno T, Kim B-Y, Park M-S (2020). Sex differences in gut microbiota. World J Mens Health.

[79] Zhao L, Wang G, Siegel P, He C, Wang H, Zhao W (2013). Quantitative genetic background of the host influences gut microbiomes in chickens. Sci Rep.

[80] Reese AT, Dunn RR (2018). Drivers of microbiome biodiversity: a review of general rules, feces, and ignorance. MBio.

[81] Valdebenito JO, Halimubieke N, Lendvai ÁZ, Figuerola J, Eichhorn G, Székely T (2021). Seasonal variation in sex-specific immunity in wild birds. Sci Rep.

[82] Dillon RJ, Vennard CT, Buckling A, Charnley AK (2005). Diversity of locust gut bacteria protects against pathogen invasion. Ecol.

[83] Benskin CMcWH, Wilson K, Jones K, Hartley IR (2009). Bacterial pathogens in wild birds: a review of the frequency and effects of infection. Biology.

[84] Shkoporov AN, Efimov BA, Kondova I, Ouwerling B, Chaplin AV, Shcherbakova VA (2016). *Peptococcus simiae* sp. Nov., isolated from rhesus macaque faeces and emended description of the genus Peptococcus. Int J Syst Evol.

[85] Zhao JB, Liu P, Huang CF, Liu L, Li EK, Zhang G (2018). Effect of wheat bran on apparent total tract digestibility, growth performance, fecal microbiota and their metabolites in growing pigs. Anim Feed Sci Technol.

[86] Sergio F, Tanferna A, Blas J, Blanco G, Hiraldo F (2019). Reliable methods for identifying animal deaths in GPS- and satellite-tracking data: review, testing, and calibration. J Appl Ecol.

[87] Pakkala JJ, Ryan Norris D, Newman AEM (2013). An experimental test of the capture-restraint protocol for estimating the acute stress response. Physiol Biochem Zool.

[88] Lynn SE, Porter AJ (2008). Trapping initiates stress response in breeding and non-breeding house sparrows *Passer domesticus*: implications for using unmonitored traps in field studies. J Avian Biol.

[89] Kesler DC, Raedeke AH, Foggia JR, Beatty WS, Webb EB, Humburg DD (2014). Effects of satellite transmitters on captive and wild mallards. Wildl Soc Bull.

[90] Gessaman JA, Nagy KA (1988). Transmitter loads affect the flight speed and metabolism of homing pigeons. Condor.

[91] Brochet A-L, van den Bossche W, Jbour S, Ndang’Ang’A PK, Jones VR, Abdou WALI (2016). Preliminary assessment of the scope and scale of illegal killing and taking of birds in the Mediterranean. Bird Conserv Int.

[92] Buechley ER, Oppel S, Efrat R, Phipps WL, Carbonell Alanís I, Álvarez E (2021). Differential survival throughout the full annual cycle of a migratory bird presents a life-history trade-off. J Anim Ecol.

