# Supplementary material for: Linking migration and microbiota at a major stopover site in a long-distance avian migrant
Source: Mov Ecol. 2022 Nov 7;10:46. doi: 10.1186/s40462-022-00347-0 (PMC9641824; doi:10.1186/s40462-022-00347-0)

**Supplementary information for:** *Linking whole migration and microbiota at a major stopover site in a long-distance avian migrant*

Nikki Thie, Ammon Corl, Sondra Turjeman, Ron Efrat, Pauline L. Kamath, Wayne M. Getz, Rauri C.K. Bowie, Ran Nathan

**Supplementary Table 1:** Results of comparing capture date, survival and post-Eilat migration distance between sex and age. Data is shown as mean ± standard deviation and sample size (*n*) between brackets, whereas statistical results are shown with statistical value and *p*-value (adjusted with Benjamini-Hochberg). Used statistical tests are indicated with numbers after the *p*-values; (1) Scheier-Ray-Hare test, (2) Person’s Chi-squared test and (3) Two-way ANOVA. Adjusted *p*-values of <0.05 are considered significant.

| **Sex** | | | | |
| --- | --- | --- | --- | --- |
| **Parameter** | **Females** | **Males** | **stat** | ***p_adj_*** |
| Capture date | 107.6 ± 8.3 (36) | 108.9 ± 6.6 (18) | (*H*) 3.026 | 0.123^1^ |
| Survival | 16/27 | 3/7 | (*X^2^*) 0.478 | 0.725^2^ |
| Post-Eilat migration distance (x 1000 km) | 3.29 ± 1.61 (16) | 5.36 ± 2.01 (3) | (*F*) 6.002 | 0.078^3^ |
| **Age** | | | | |
| **Parameter** | **Juveniles** | **Adults** | **stat** | ***p_adj_*** |
| Capture date | 108.1 ± 8.1 (23) | 107.7 ± 7.7 (31) | (*H*) 1.518 | 0.327^1^ |
| Survival | 7/10 | 12/24 | (*X^2^*) 0.478 | 0.490^2^ |
| Post-Eilat migration distance (x 1000 km) | 2.31 ± 0.94 (7) | 4.38 ± 1.74 (12) | (*F*) 10.009 | 0.018^3^ |

**Supplementary Table 2:** Results from model selection explaining variation in capture date and post-Eilat migration distance. All included models are shown, ranked by AICc values.

| **Model** | **Explanatory variables** | **df** | **Log Link** | **AICc** | **ΔAICc** | **weight** |
| --- | --- | --- | --- | --- | --- | --- |
| **Capture date (females)** | | | | | | |
| **1** | **SMI** | **3** | **-48.901** | **104.552** | **0.000** | **0.578** |
| 2 | SMI:age | 4 | -48.801 | 106.892 | 2.340 | 0.179 |
| 3 | SMI + age | 4 | -48.855 | 107.000 | 2.449 | 0.170 |
| 4 | NULL | 2 | -52.170 | 108.704 | 4.152 | 0.073 |
| **Capture date (males)** | | | | | | |
| **1** | **NULL** | **2** | **-21.823** | **48.446** | **0.000** | **0.704** |
| 2 | SMI | 3 | -21.823 | 51.360 | 2.914 | 0.164 |
| 3 | SMI:age | 4 | -20.989 | 53.054 | 4.608 | 0.070 |
| 4 | SMI + age | 4 | -21.128 | 53.333 | 4.886 | 0.061 |
| **Post-Eilat migration distance (females)** | | | | | | |
| **1** | **SMI:age** | **4** | **-16.256** | **44.149** | **0.000** | **0.419** |
| **2** | **SMI + age** | **4** | **-16.521** | **44.679** | **0.530** | **0.322** |
| **3** | **NULL** | **2** | **-20.467** | **45.858** | **1.708** | **0.178** |
| 4 | SMI | 3 | -19.717 | 47.435 | 3.285 | 0.081 |

**Supplementary Table 3:** Most abundant (i.e. prevalence >80%) genera across all (*n*=47) steppe buzzard samples with corresponding mean relative abundance.

| Genus | Prevalence (%) | Mean relative abundance (% ± s.d.) |
| --- | --- | --- |
| *Bacteroides* | 100.0 | 2.6 ± 3.5 |
| *Clostridium sensu stricto 1* | 100.0 | 9.2 ± 12.8 |
| *Corynebacterium* | 100.0 | 21.5 ± 22.8 |
| *Enterococcus* | 100.0 | 2.7 ± 3.8 |
| *Escherichia-Shigella* | 100.0 | 7.3 ± 13.1 |
| *Fusobacterium* | 100.0 | 2.7 ± 3.4 |
| *Lactobacillus* | 100.0 | 0.1 ± 0.0 |
| *Paeniclostridium* | 100.0 | 4.6 ± 9.2 |
| *Peptostreptococcus* | 100.0 | 4.3 ± 4.5 |
| *Staphylococcus* | 100.0 | 1.8 ± 3.5 |
| *Varibaculum* | 100.0 | 9.8 ± 8.0 |
| *Actinomyces* | 97.9 | 0.9 ± 1.6 |
| *Catellicoccus* | 97.9 | 0.4 ± 1.7 |
| *Chelonobacter* | 97.9 | 0.1 ± 0.1 |
| *Peptoniphilus* | 97.9 | 1.0 ± 1.7 |
| *Suttonella* | 97.9 | 0.1 ± 0.2 |
| *Megamonas* | 93.6 | 0.3 ± 0.7 |
| *Fastidiosipila* | 91.5 | 1.8 ± 3.9 |
| *Aerococcus* | 89.4 | 0.9 ± 2.2 |
| *Campylobacter* | 87.2 | 0.3 ± 0.4 |
| *Porphyromonas* | 85.1 | 2.0 ± 4.2 |
| *Sutterella* | 85.1 | 1.0 ± 1.3 |
| *Murdochiella* | 83.0 | 0.4 ± 1.1 |
| *Mycoplasma* | 80.9 | 2.2 ± 4.1 |
| *S5-A14a* | 80.9 | 0.4 ± 0.8 |

**Supplementary Table 4:** Prevalence (%), relative abundance ± standard deviation and classification (phylum, class, family, genus, species) for the 27 most prevalent ASVs (>80%) found in the steppe buzzard microbiome dataset.

| ASV # | prev. (%) | mean ± s.d. | Phylum | Class | Family | Genus | Species |
| --- | --- | --- | --- | --- | --- | --- | --- |
| 182 | 100 | 6.9 ± 12.4 | Proteobacteria | Gammaproteobacteria | Enterobacteriaceae | *Escherichia-Shigella* | *coli* |
| 332 | 100 | 0.0 ± 0.0 | Firmicutes | Bacilli | Staphylococcaceae | *Staphylococcus* | *saprophyticus* |
| 727 | 100 | 2.2 ± 3.4 | Firmicutes | Bacilli | Enterococcaceae | *Enterococcus* | *faecium* |
| 731 | 100 | 0.0 ± 0.0 | Firmicutes | Bacilli | Lactobacillaceae | *Lactobacillus* | NA |
| 902 | 100 | 5.0 ± 7.2 | Firmicutes | Clostridia | Clostridiaceae | *Clostridium sensu stricto 1* | *perfringens* |
| 1180 | 100 | 4.0 ± 7.9 | Firmicutes | Clostridia | Peptostreptococcaceae | *Paeniclostridium* | *sordellii* |
| 333 | 97.9 | 0.0 ± 0.0 | Firmicutes | Bacilli | Staphylococcaceae | *Staphylococcus* | *sciuri* |
| 806 | 95.7 | 0.0 ± 0.0 | Proteobacteria | Gammaproteobacteria | Pasteurellaceae | *Chelonobacter* | *oris* |
| 818 | 95.7 | 0.0 ± 0.0 | Proteobacteria | Gammaproteobacteria | Moraxellaceae | *NA* | NA |
| 520 | 93.6 | 0.0 ± 0.0 | Proteobacteria | Gammaproteobacteria | Cardiobacteriaceae | *Suttonella* | *ornithocola* |
| 1017 | 93.6 | 0.0 ± 0.0 | Actinobacteriota | Actinobacteria | Corynebacteriaceae | *Corynebacterium* | NA |
| 1231 | 93.6 | 0.0 ± 0.0 | Firmicutes | Clostridia | Family XI | *Peptoniphilus* | NA |
| 419 | 91.5 | 0.0 ± 0.0 | Firmicutes | Clostridia | Peptostreptococcaceae | *Peptostreptococcus* | NA |
| 817 | 91.5 | 0.0 ± 0.0 | Proteobacteria | Gammaproteobacteria | Moraxellaceae | *Moraxella* | NA |
| 1077 | 91.5 | 4.1 ± 5.7 | Actinobacteriota | Actinobacteria | Actinomycetaceae | *Varibaculum* | NA |
| 1083 | 91.5 | 0.0 ± 0.0 | Firmicutes | Clostridia | Family XI | *NA* | NA |
| 837 | 89.4 | 0.0 ± 0.0 | Proteobacteria | Gammaproteobacteria | Pasteurellaceae | *Chelonobacter* | *oris* |
| 1012 | 89.4 | 0.0 ± 0.1 | Actinobacteriota | Actinobacteria | Corynebacteriaceae | *Corynebacterium* | *aquilae* |
| 1210 | 89.4 | 2.8 ± 3.2 | Firmicutes | Clostridia | Peptostreptococcaceae | *Peptostreptococcus* | NA |
| 339 | 87.2 | 1.3 ± 2.7 | Firmicutes | Bacilli | Staphylococcaceae | *Staphylococcus* | aureus |
| 660 | 87.2 | 0.6 ± 1.3 | Firmicutes | Bacilli | Aerococcaceae | *Aerococcus* | *urinaehominis* |
| 717 | 87.2 | 0.0 ± 0.0 | Firmicutes | Bacilli | Catellicoccaceae | *Catellicoccus* | NA |
| 811 | 87.2 | 0.0 ± 0.0 | Proteobacteria | Gammaproteobacteria | Neisseriaceae | *Neisseria* | NA |
| 838 | 87.2 | 0.0 ± 0.0 | Proteobacteria | Gammaproteobacteria | Pasteurellaceae | *Chelonobacter* | *oris* |
| 659 | 85.1 | 0.0 ± 0.0 | Firmicutes | Negativicutes | Selenomonadaceae | *Megamonas* | NA |
| 1198 | 83.0 | 1.0 ± 1.7 | Firmicutes | Clostridia | Peptostreptococcaceae | *Peptostreptococcus* | *anaerobius* |
| 634 | 80.9 | 0.0 ± 0.0 | Firmicutes | Bacilli | Bacillaceae | *Virgibacillus* | NA |

**Supplementary Table 5:** Results from microbiota comparisons between females (*n*=34) and males (*n*=13), and juveniles (*n*=20) and adults (*n*=27). α-diversity measures, Shannon diversity, Chao1 index and Faith’s PD, are shown as mean ± standard deviation and include *F*-statistic and p-value from two-way ANOVA used for the comparisons. Comparison of homogeneity of variance (*F*-statistic and *p*-value) and community dissimilarity (R^2^, *F*-statistic and *p*-value) are shown for the *β*-diversity measures Unifrac, Weighted Unifrac, Jaccard, and Bray-Curtis. For both α-diversity and *β*-diversity comparisons, *p*-values < 0.05 indicate significant differences. Significantly abundant genera as indicated by ANCOM-BC are shown with W-, *p*- and *q*-values (*p_adj_*).

|  | **Sex** | | | | **Age** | | | |
| --- | --- | --- | --- | --- | --- | --- | --- | --- |
| ***α*-diversity** | **Females** | **Males** | ***F*** | ***p*** | **Juveniles** | **Adults** | ***F*** | ***p*** |
| Shannon [log] | 2.84 ± 0.58 | 2.96 ± 0.68 | 0.413 | 0.524 | 2.77 ± 0.54 | 2.94 ± 0.65 | 1.289 | 0.262 |
| Chao1 | 262.8 ± 58.0 | 269.1 ± 59.7 | 0.108 | 0.744 | 264.0 ± 47.7 | 265.0 ± 65.3 | 0.000 | 0.993 |
| Faith’s PD [inv] | 26.45 ± 6.40 | 28.79 ± 6.68 | 1.507 | 0.226 | 27.21 ± 7.57 | 27.01 ± 5.72 | 0.001 | 0.970 |
| ***β*-diversity** |  | ***R^2^*** | ***F*** | ***p*** |  | ***R^2^*** | ***F*** | ***p*** |
| Unifrac | Dissimilarity | 4.2% | 1.953 | 0.006 | Dissimilarity | 2.7% | 1.269 | 0.136 |
|  | Homogeneity |  | 0.455 | 0.509 | Homogeneity |  | 0.052 | 0.812 |
| Weighted Unifrac | Dissimilarity | 5.9% | 2.830 | 0.011 | Dissimilarity | 3.4% | 1.645 | 0.123 |
|  | Homogeneity |  | 0.115 | 0.742 | Homogeneity |  | 1.479 | 0.236 |
| Jaccard | Dissimilarity | 4.2% | 1.989 | 0.010 | Dissimilarity | 3.0% | 1.411 | 0.083 |
|  | Homogeneity |  | 2.399 | 0.138 | Homogeneity |  | 0.892 | 0.356 |
| Bray-Curtis | Dissimilarity | 5.3% | 2.553 | 0.011 | Dissimilarity | 3.6% | 1.706 | 0.074 |
|  | Homogeneity |  | 2.976 | 0.092 | Homogeneity |  | 1.187 | 0.281 |
| **Abundances** | **Genus** | ***W*** | ***p*** | ***q*** | **Genus** | ***W*** | ***p*** | ***q*** |
|  | *Oceanivirga* | 4.488 | <0.001 | 0.001 | *Arcanobacterium* | 2.289 | <0.001 | <0.001 |
|  | *Campylobacter* | 3.996 | <0.001 | 0.003 | *Negativicoccus* | 1.912 | <0.001 | <0.001 |
|  | *Mycoplasma* | 3.788 | <0.001 | 0.005 | *Salmonella* | 1.763 | <0.001 | <0.001 |
|  | *Veillonella* | 3.611 | <0.001 | 0.006 |  |  |  |  |
|  | *Desulfovibrio* | -1.720 | <0.001 | 0.000 |  |  |  |  |
|  | *Ercella* | -1.860 | <0.001 | 0.000 |  |  |  |  |
|  | *Paraeggerthella* | -1.935 | <0.001 | 0.000 |  |  |  |  |
|  | *Sarcina* | -2.018 | <0.001 | 0.000 |  |  |  |  |
|  | *Jonquetella* | -2.743 | <0.001 | 0.000 |  |  |  |  |
|  | *Negativicoccus* | -3.090 | <0.001 | 0.000 |  |  |  |  |
|  | *Anaerococcus* | -3.130 | <0.001 | 0.000 |  |  |  |  |
|  | *Arcanobacterium* | -3.620 | <0.001 | 0.000 |  |  |  |  |

**Supplementary Table 6:** Results from microbiota comparisons between female individuals that did (“survive”, *n*=14) and did not (“dead”, *n*=11) survive full post-Eilat spring migration. α-diversity measures, Shannon diversity, Chao1 index and Faith’s PD, are shown as mean ± standard deviation and include *t*-statistic, df and *p*-value from Student’s T-test used for the comparisons. Comparison of homogeneity of variance (*F*-statistic and *p*-value) and community dissimilarity (R^2^, *F*-statistic and *p*-value) are shown for the *β*-diversity measures Bray-Curtis and Weighted Unifrac. For both α-diversity and *β*-diversity comparisons, *p*-values < 0.05 indicate significant differences.

| ***α*-diversity** | **Survive** | **Dead** | ***t*** | **df** | ***p*** |
| --- | --- | --- | --- | --- | --- |
| Shannon | 2.80 ± 0.65 | 3.08 ± 0.46 | -1.271 | 22.84 | 0.217 |
| Chao1 | 275.7 ± 69.0 | 259.8 ± 54.0 | 0.605 | 23.00 | 0.552 |
| Faith’s PD | 27.7 ± 7.7 | 45.6 ± 4.0 | -1.328 | 22.99 | 0.197 |
| ***β*-diversity** |  | ***R^2^*** |  | ***F_1,23_*** | ***p*** |
| Unifrac | Dissimilarity | 4.2% |  | 0.989 | 0.427 |
|  | Homogeneity |  |  | 1.026 | 0.325 |
| Weighted Unifrac | Dissimilarity | 3.9% |  | 0.862 | 0.509 |
|  | Homogeneity |  |  | 1.558 | 0.219 |
| Jaccard | Dissimilarity | 4.8% |  | 1.159 | 0.208 |
|  | Homogeneity |  |  | 2.070 | 0.162 |
| Bray-Curtis | Dissimilarity | 5.2% |  | 1.159 | 0.228 |
|  | Homogeneity |  |  | 2.173 | 0.153 |

**Supplementary Results 1**:

We investigated whether there were any microbial differences between samples from the final dataset that came from one extraction (*n*=44) or combined extractions (*n*=3). The two groups of extractions did not differ significantly in α-diversity measured by Shannon (Student’s T-test on log-transformed data: *t*=0.238, df=2.88, *p=*0.828; **Figure S1a**), Chao1 index (Student’s T-test on log-transformed data: *t*=2.192, df=2.31, *p=*0.142; **Figure S1b**) or Faith’s PD (Student’s T-test on inverse-transformed data: *t*=0.573, df=2.23, *p=*0.619; **Figure S1c**). Additionally, the samples from combined extractions did not stand out upon plotting the most abundant phyla (**Figure S1d**) or ordination plots of community compositions based on four different distance metrices: Unifrac, Weighted Unifrac, Jaccard and Bray-Curtis (**Figure S1e,f,g,h**).

**Supplementary Figure 1**:

Boxplots for (a) Shannon diversity, (b) Chao1 diversity index, and (b) Faith’s PD, of steppe buzzard samples from one extraction (*n*=44, purple) or combined extractions (*n*=3, pink). (d) Relative abundance of the most common phyla (relative abundance of >1%) per sample, separated by one extraction (left) or combined extractions (right). (e) MDS (multidimensional scaling) plots of Unifrac distances and (f) Weighted Unifrac distances, (g) Jaccard distances and (h) Bray-Curtis distances colored by extraction (one extraction in purple and combined extractions in pink)


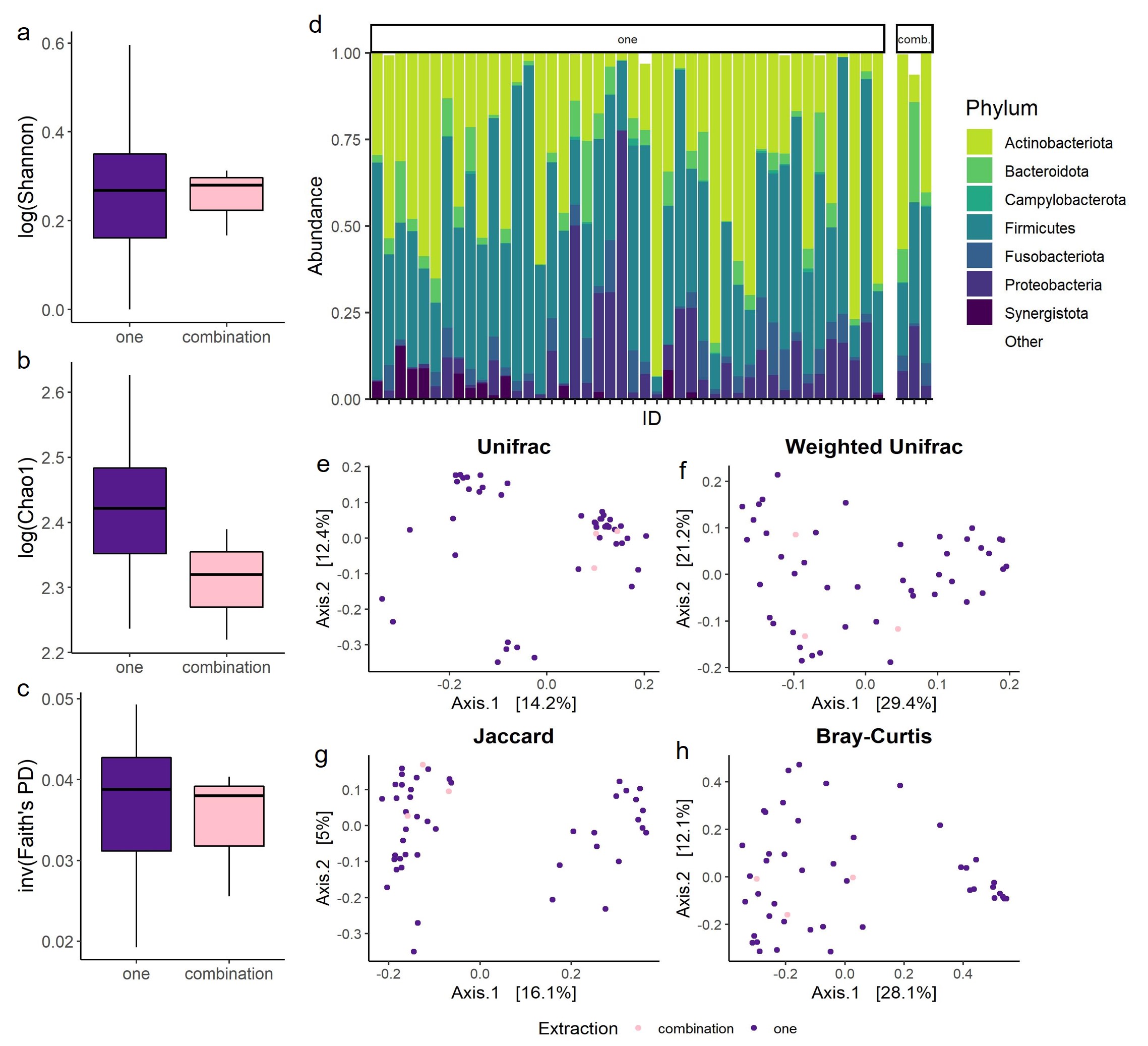


**
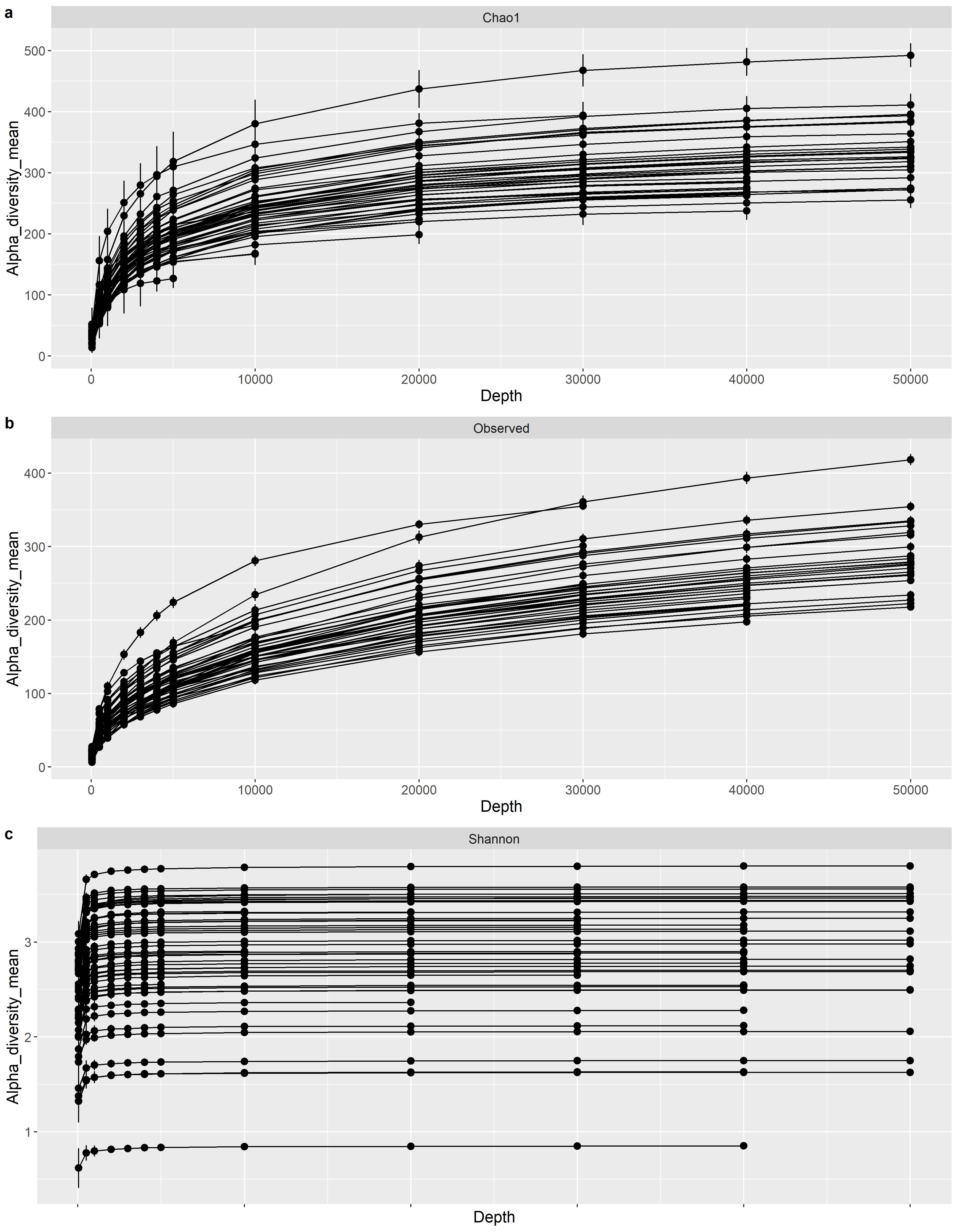
Supplementary Figure 2**: Rarefaction curves for all microbiome samples (n=47), for three α-diversity measures: a) Chao1 index, b) Observed diversity, and c) Shannon diversity index.

**
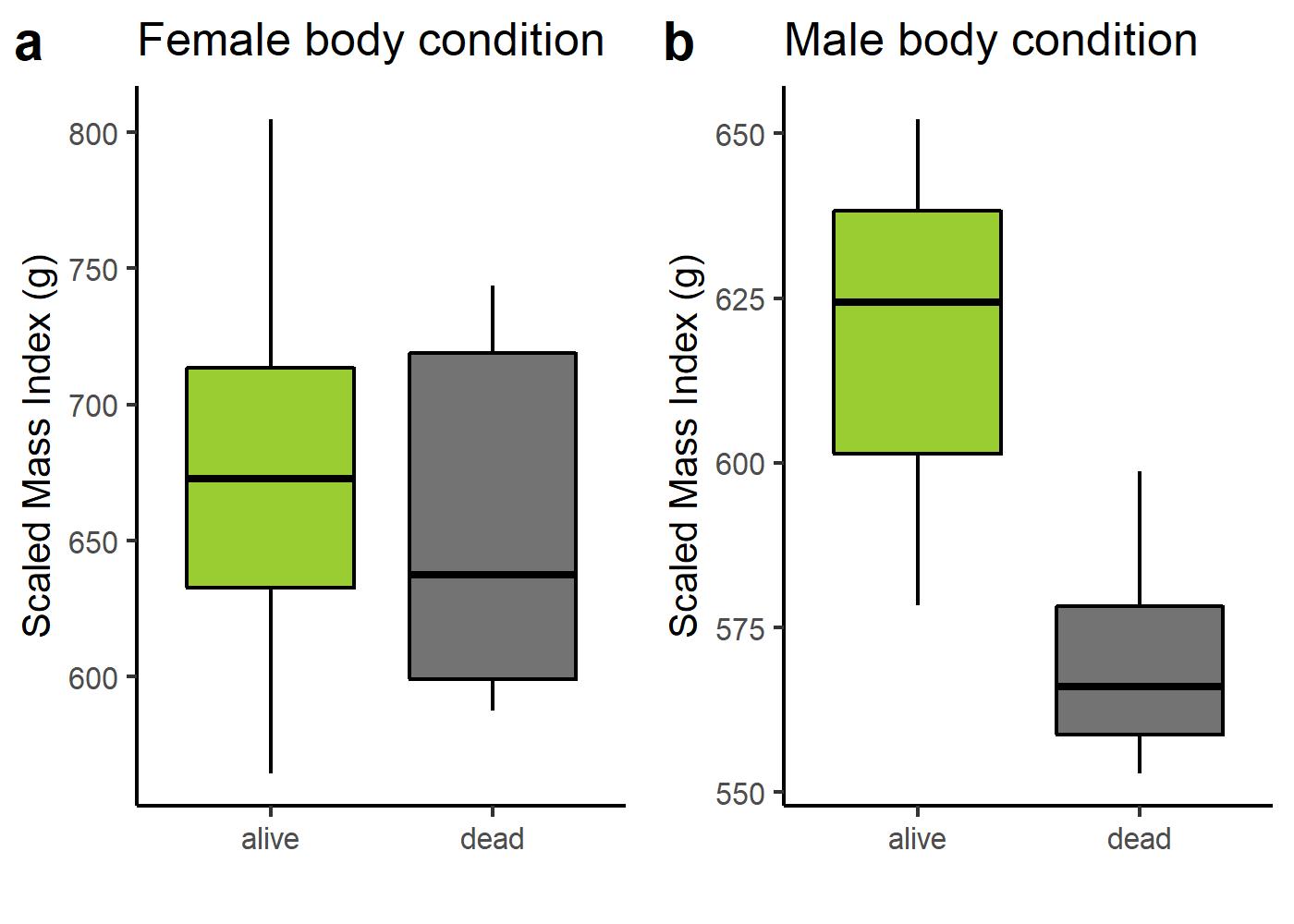
Supplementary Figure 3**: Boxplots comparing the body condition of surviving (alive, green) vs. non-surviving (dead, grey) female (a) and male (b) steppe buzzards.

**Supplementary Figure 4**: **Comparison of male and female steppe buzzard microbiota composition**

(a) MDS (multidimensional scaling) plots Jaccard distances and (b) Bray-Curtis distances colored by sex (males in purple, females in green), including ellipses (dashed lines) of 95% confidence around centroids (⊕).

**
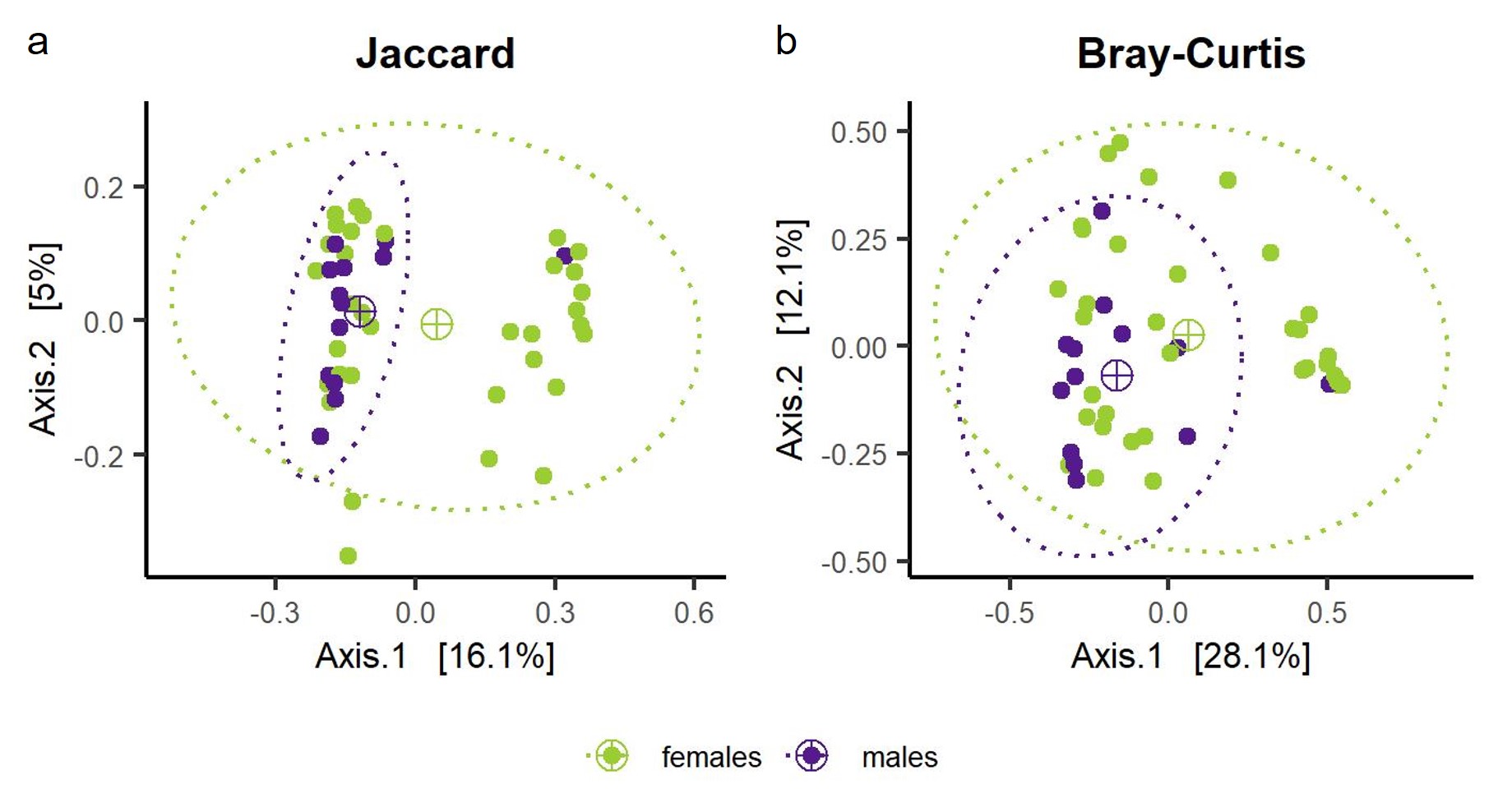
**

**Supplementary Figure 5**: **Association between capture date and microbiota β-diversity in female steppe buzzards**

(a) MDS (multidimensional scaling) plots Jaccard distances and (b) Bray-Curtis distances colored capture date, ranging from purple (earlier arrival) to yellow (later arrival).

**
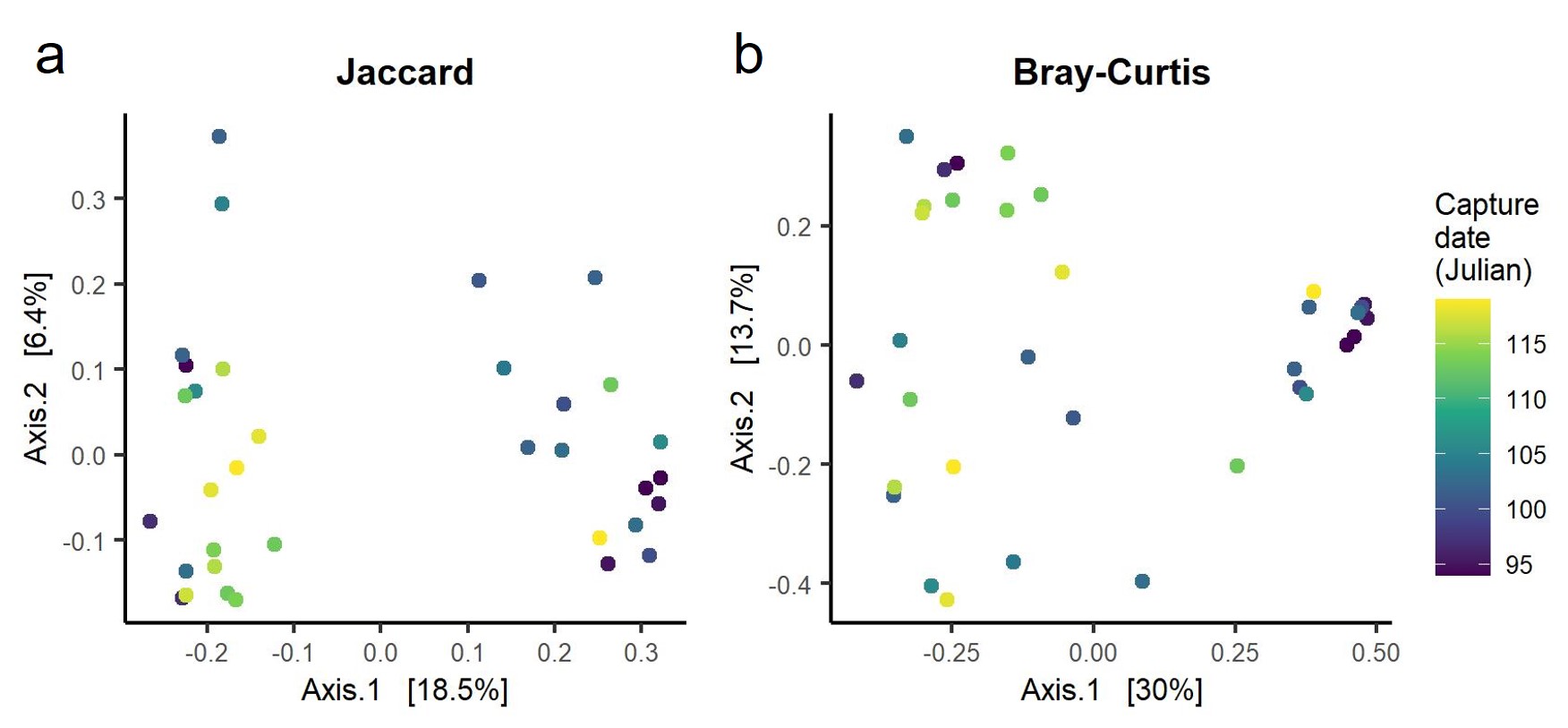
**

**Supplementary Figure 6**: **Association between survival and microbiota β-diversity in female steppe buzzards**

(a) MDS (multidimensional scaling) plots Jaccard distances and (b) Bray-Curtis distances colored by female survival (alive in green, dead in grey), including ellipses (dashed lines) of 95% confidence around centroids (⊕).

**
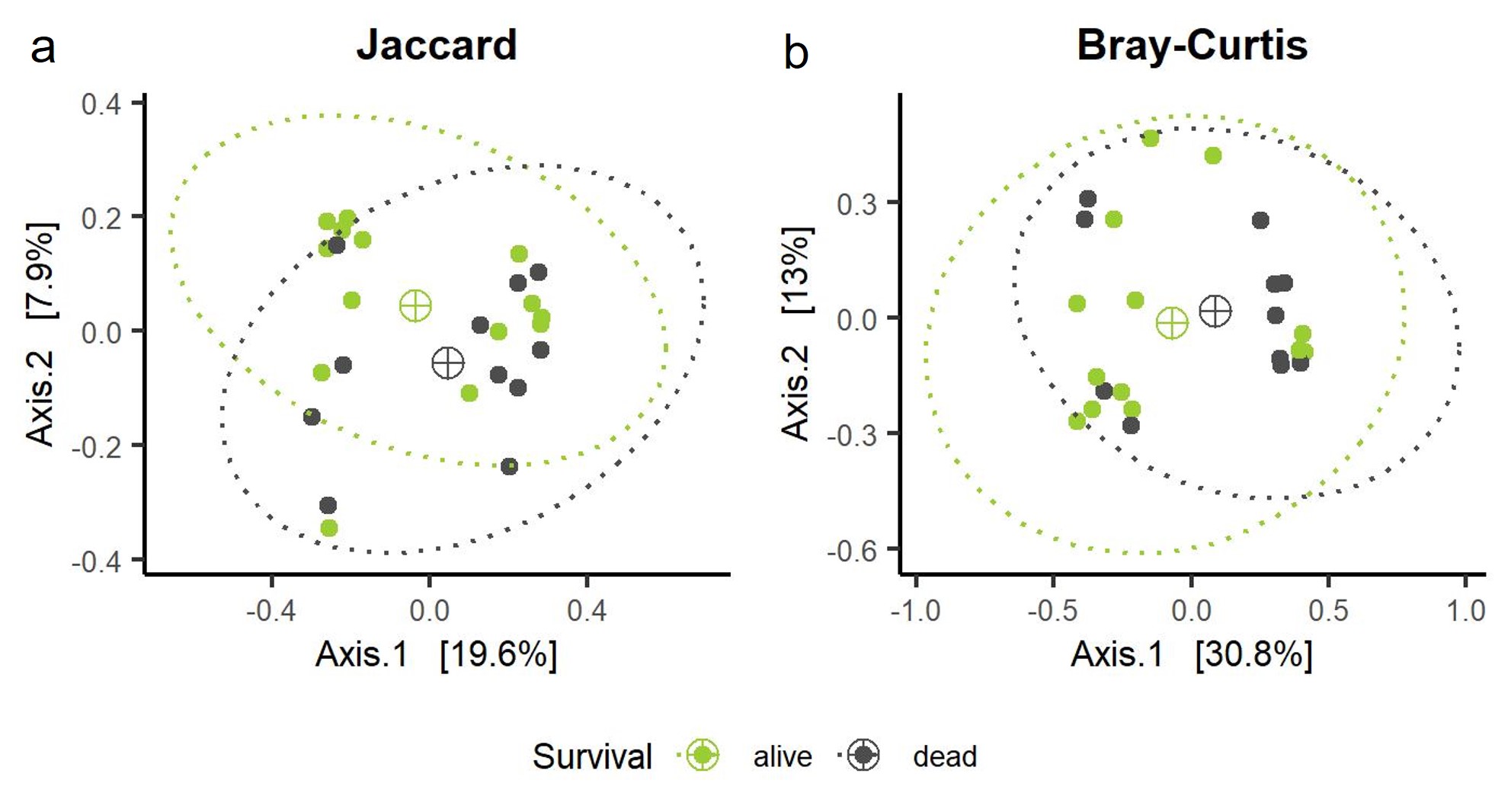
**

**Supplementary Results 2**:

*Body condition vs. microbiome*

Male (*n*=13) body condition (SMI) was not related to Shannon α-diversity index (*R^2^*=0.18, *F*_2,10_=1.081, *p*=0.376), Chao1 index [log transf] (*R^2^*=0.10, *F*­_2,10_=0.534, *p*=0.602) or Faith’s PD (*R^2^*=0.25, *F*­_2,10_=1.649, *p*=0.241). There was no significant influence of male body condition on community composition in Unifrac (*R*^2^=0.09, *F*_1,11_=1.097, *p*=0.300), Jaccard (*R*^2^=0.10, *F*_1,11_=1.201, *p*=0.165) or Bray-Curtis (*R^2^*=0.12, *F*_1,11_=1.437, *p*=0.109), but there was a trend for Weighted Unifrac (*R^2^*=0.14, *F*_1,11_=1.786, *p*=0.086). However, no phyla or genera were found to be differentially abundant (ANCOM-BC) with increasing or decreasing body condition.

*Capture date vs. microbiome*

For males (*n*=13), there were no correlations of capture date with either Shannon diversity index [inv transf] (*R^2^*=0.01, *F*­_2,10_=0.071, *p*=0.932), Chao1 index [log transf] (*R^2^*=0.05, *F*_2,10_=0.249, *p*=0.784) or Faith’s PD (*R^2^*=0.20, *F*­_2,10_=1.223, *p*=0.335). Capture date was correlated with community composition for Weighted Unifrac (*R^2^*=0.18, *F*_1,11_=2.375, *p*=0.026; **Figure S7b**), Jaccard (*R^2^*=0.13, *F*_1,11_=1.656, *p*=0.012; **Figure S7c**), and Bray-Curtis (*R^2^*=0.15, *F*_1,11_=2.016, *p*=0.014; **Figure S7d**), but not for Unifrac (*R^2^*=0.09, *F*_1,11_=1.136, *p*=0.242; **Figure S7a**). Specifically, there were higher prevalences and abundances of the genera *Gemella*, *Peptococcus*, *Globicatella*, *Neisseria*, *Savagea* and *Dubosiella* among early arrivals, whereas the genera *Staphylococcus* was increased in later arrivals (ANCOM-BC: **Figure S7c**).

Since we only tagged a total of 7 males, of which 1 individual lost connection, 2 survived spring migration and 4 died before reaching the breeding grounds, we do not have a large enough sample size to test body condition and microbiota differences with survival.

**
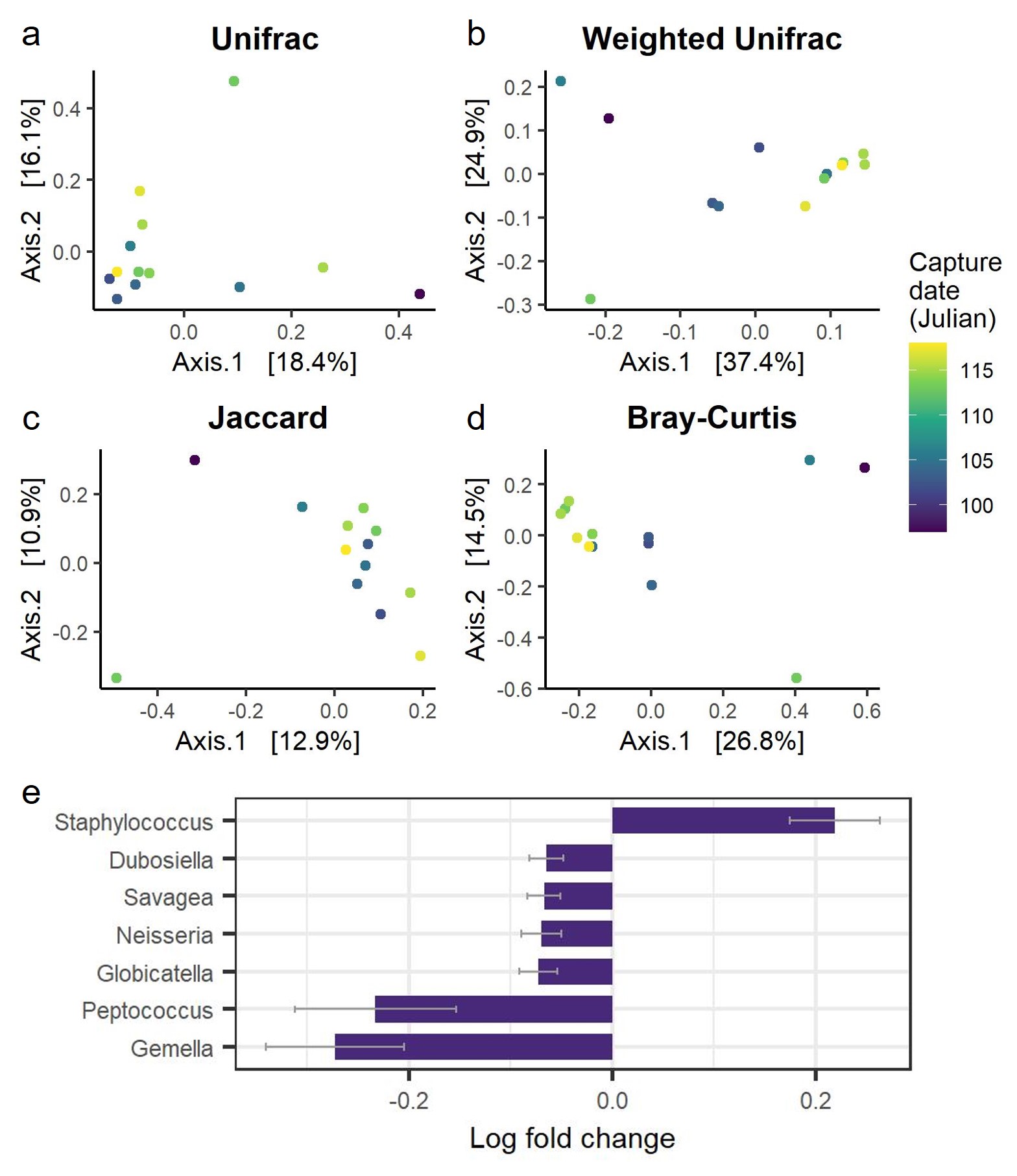
Supplementary Figure 7**: MDS (multidimensional scaling) ordination plots for (a) Unifrac distances, (b) Weighted Unifrac distances, (c) Jaccard distances, and (d) Bray-Curtis distances of males (*n*=13), with arrival date colored from purple (early) to yellow (later). (e) Differentially abundant genera as determined by ANCOM-BC analysis, with positive log-fold change values indicating the taxa increased with later arrival date and negative log-fold change values indicate a that the taxa decreased with later arrival date.

**Supplementary Figure 8**: Migratory routes (+ route fidelity) from three individuals that have been tracked over at least two subsequent years, starting with capture in Eilat during spring migration in 2019. Breeding and wintering locations are indicated by open circles and corresponding years. Spring migrations are colored in green, autumn migration in orange. Demographics of the birds are (a) female captured as second year individual (EURING age 7) in 2019, (b) male captured as mature individual (EURING age 8) in 2019, and (c) male captured as first year individual (EURING age 5) in 2019.


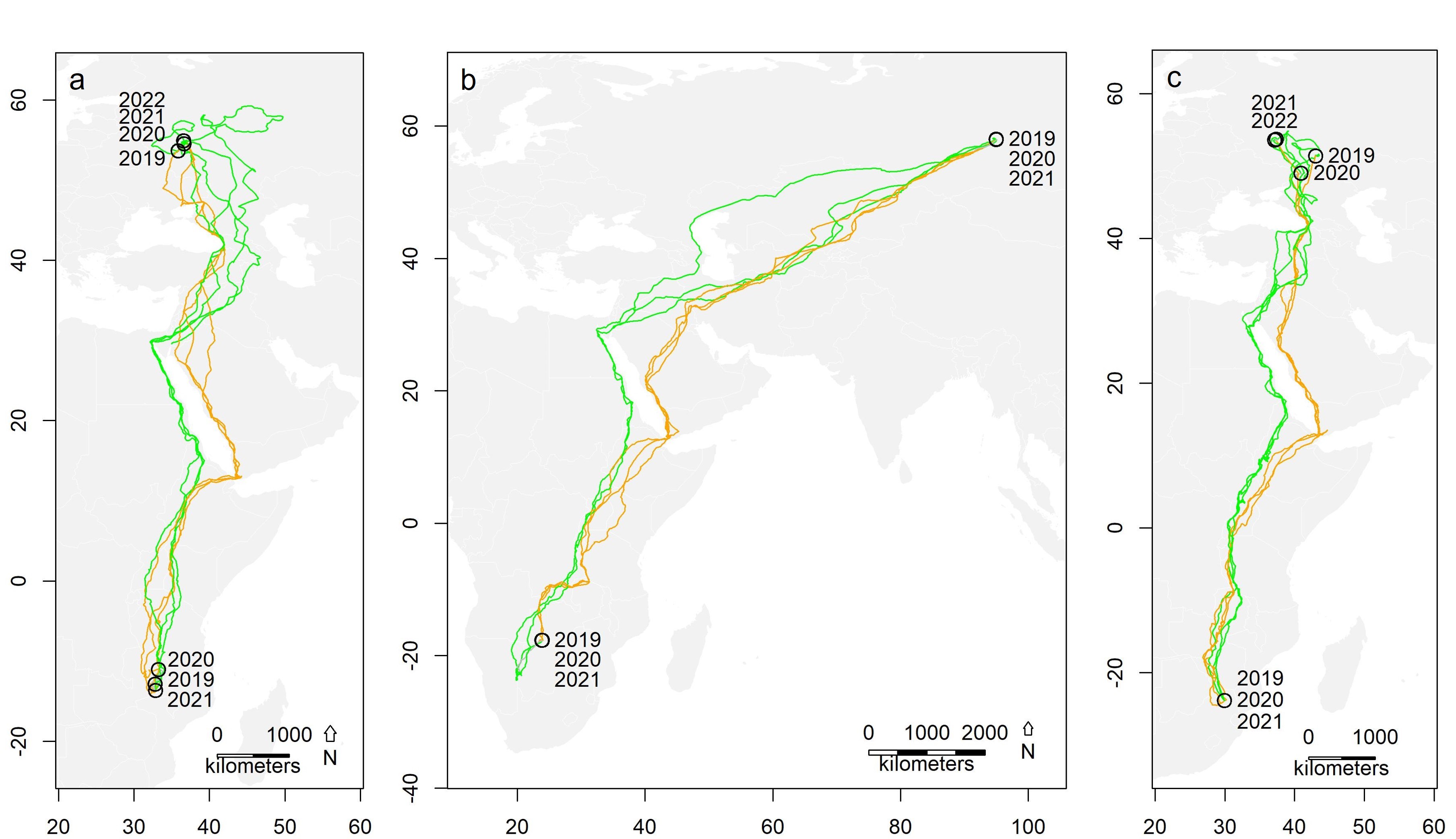

Supplement: Supplementary file 1 — Additional file 1. Supplementary results, tables, and figures. [file 40462_2022_347_MOESM1_ESM.docx]
